# STAT3 blockade ameliorates LPS-induced kidney injury through macrophage-driven inflammation

**DOI:** 10.1186/s12964-024-01841-1

**Published:** 2024-10-04

**Authors:** Song-Hee Lee, Kyu Hong Kim, Seong Min Lee, Seong Joon Park, Sunhwa Lee, Ran-Hui Cha, Jae Wook Lee, Dong Ki Kim, Yon Su Kim, Sang-Kyu Ye, Seung Hee Yang

**Affiliations:** 1 Department of Biomedical Sciences, Seoul National University, Seoul, Republic of Korea; 2 Department of Pharmacology, Seoul National University, Seoul, Republic of Korea; 3 Department of Internal Medicine, Division of Nephrology, Kangwon National University Hospital, Chuncheon, Gangwon-Do Republic of Korea; 4 Nephrology Clinic, National Cancer Center of Korea, Seoul, Republic of Korea; 5 Department of Internal Medicine, Seoul National University Hospital, Seoul, Republic of Korea; 6 Department of Internal Medicine, Seoul National University College of Medicine, Seoul, Republic of Korea; 7 Department of Kidney Research Institute, Seoul National University Medical Research Center, Seoul, Republic of Korea; 8 Biomedical Research Institute, Seoul National University Hospital, Hospital, Seoul, Republic of Korea

**Keywords:** STAT3, LPS, AKI, Macrophage-driven inflammation, Fibrosis

## Abstract

**Background:**

Signal transducer and activator of transcription 3 (STAT3), a multifaceted transcription factor, modulates host immune responses by activating cellular response to signaling ligands. STAT3 has a pivotal role in the pathophysiology of kidney injury by counterbalancing resident macrophage phenotypes under inflammation conditions. However, STAT3’s role in acute kidney injury (AKI), particularly in macrophage migration, and in chronic kidney disease (CKD) through fibrosis development, remains unclear.

**Methods:**

Stattic (a JAK2/STAT3 inhibitor, 5 mg/kg or 10 mg/kg) was administered to evaluate the therapeutic effect on LPS-induced AKI (L-AKI) and LPS-induced CKD (L-CKD), with animals sacrificed 6–24 h and 14 days post-LPS induction, respectively. The immune mechanisms of STAT3 blockade were determined by comparing the macrophage phenotypes and correlated with renal function parameters. Also, the transcriptomic analysis was used to confirm the anti-inflammatory effect of L-AKI, and the anti-fibrotic role was further evaluated in the L-CKD model.

**Results:**

In the L-AKI model, sequential increases in BUN and blood creatinine levels were time-dependent, with a marked elevation of 0–6 h after LPS injection. Notably, two newly identified macrophage subpopulations (CD11b^high^F4/80^low^ and CD11b^low^F4/80^high^), exhibited population changes, with an increase in the CD11b^high^F4/80^low^ population and a decrease in the CD11b^low^F4/80^high^ macrophages. Corresponding to the FACS results, the tubular injury score, NGAL, F4/80, and p-STAT3 expression in the tubular regions were elevated. STAT3 inhibitor injection in L-AKI and L-CKD mice reduced renal injury and fibrosis. M2-type subpopulation with CD206 in CD11b^low^F4/80^high^ population increased in the Stattic-treated group compared with that in the LPS-alone group in the L-AKI model. Additionally, STAT3 inhibitor reduced inflammation driven by LPS-stimulated macrophages and epithelial cells injury in the co-culture system. Transcriptomic profiling identified 3 common genes in the JAK-STAT, TLR, and TNF signaling pathways and 11 common genes in the LPS with macrophage response. The PI3K-AKT (*IL-6*, *Akt3*, and *Pik3r1*) and JAK-STAT pathways were determined as potential Stattic targets. Further confirmation through mRNA and protein expressions analyses showed that Stattic treatment reduced inflammation in the L-AKI and fibrosis in the L-CKD mice.

**Conclusions:**

STAT3 blockade effectively mitigated inflammation by retrieving the CD11b^low^F4/80^high^ population, further emphasizing the role of STAT3-associated macrophage-driven inflammation in kidney injury.

**Supplementary Information:**

The online version contains supplementary material available at 10.1186/s12964-024-01841-1.

## Abstract (plain language summary)

This study investigated the role of STAT3 in LPS-induced acute kidney injury (AKI) and its prolonged pathophysiological effect. In a mouse model, blocking STAT3 with Stattic reduced inflammation and fibrosis, decreased the levels of inflammatory and extracellular matrix (ECM) substances, reduced the number of certain immune cells (macrophages), and influenced specific genes related to inflammation. The findings suggest that targeting STAT3 is a promising approach to treat AKI and CKD by controlling the inflammation and the immune response as well as ECM accumulation. This study provides novel insights into AKI and CKD progression and will facilitate the development of new treatments for kidney injuries at various stages.

**Supplementary Information:**

The online version contains supplementary material available at 10.1186/s12964-024-01841-1.

## Background

Signal transducer and activator of transcription 3 (STAT3) is a transcription factor integral to cytokine and growth hormone receptor signaling. The JAK-STAT signaling pathway is activated upon binding external stimuli to these receptors, resulting in the phosphorylation of JAKs. Subsequently, JAKs recruit and phosphorylate STATs, which translocate to the nucleus to upregulate the expression of target genes [1]. STAT activation, orchestrated by more than 50 cytokines and growth factors [2], is made highly complex by its regulatory epigenetic mechanisms in various cellular functions [3, 4]. In vitro and in vivo transcriptomic profiling and functional studies revealed that STAT1 and STAT3 play important roles within renal cells across various renal pathologies, such as ischemia–reperfusion injury, diabetic nephropathy, and glomerulosclerosis [5–7]. However, the precise mechanisms by which STAT3 influences the inflammatory response, especially considering the diverse origins and functions of innate immune cells like macrophages in AKI, remains elusive.

STAT3 considerably influences macrophage polarization via the JAK-STAT, NF-κB, and MAPK signaling pathways. STAT3 signaling is crucial in modulating the host inflammatory response and preventing chronic inflammatory states. Determining macrophage phenotypic characteristics was closely coordinated by activating the JAK-STAT signaling pathway, which stimulated the M1-type macrophages in AKI. This stimulation is associated with the production of certain chemokines and cytokines, including TNF-α, IL-1β, IL-6, IFN-γ, and IL-12 [8]. This is relevant in kidney diseases, where macrophages, identified by the high expression of F4/80 and CD11b, can either contribute to renal protection or exacerbate injury, with their roles varying according to the specific condition of the kidney [9]*.*

Five distinct subgroups (CD11b^hi^, CD11c^hi^, CD11b^hi^, CD11c^low^, CD11b^int^, CD11b^lo^, CD11c^hi^, and CD11b^−^ CD11c^int^) of resident kidney macrophages have been identified, providing insights into the heterogeneity of macrophage responses in kidney injury and fibrosis. In ischemic AKI, for example, the CD11b^hi^ CD11c^int^ F4/80^hi^ macrophages display anti-inflammatory properties and remarkable adaptability, including the capacity for self-renewal and response to disease conditions. In contrast, CD45^+^ Ly6G^−^ F4/80^int^ CD11b^high^ macrophages offer a protective effect under ischemic conditions, highlighting the complexity of macrophage function in renal injury [10].

Lipopolysaccharide (LPS) from Gram-negative bacteria is a potent inducer of the L-AKI mouse model. In renal tubular epithelial cells, LPS triggers inflammatory cascades via toll-like receptor (TLR)-4, leading to inflammation, oxidative stress, tubular atrophy, and reduced kidney function. In macrophage experiments, LPS and IFN-γ are typically used to stimulate M1-type macrophages. These macrophages express surface receptors CD80, CD40, MHC-II, and CD86 and release proinflammatory cytokines, including TNF-α, IL-1β, IL-6, and iNOS, distinguishing them from M2-type macrophage surface markers (CD206, CD205, CD163, and Arg-1) [11–13]. Once L-AKI occurs, it increases the risk of progressing to chronic kidney disease (CKD), and the development of CKD leads to kidney fibrosis, which often results in high mortality rates [14]. Therefore, research into the treatment of such kidney diseases is critical.

Given their significant roles in the sepsis-AKI model, kidney-resident macrophages expressing F4/80^hi^ show potential anti-inflammatory capabilities and critical involvement in cellular communication, including the TLR-4 pathway [15]. The behavior of these macrophage subsets upon endotoxin exposure was investigated [16]. Moreover, we reported the mechanism of STAT3 inhibitor against kidney disease, through previous researches [17–23]. Subsequently, we established an LPS-induced chronic disease model to observe the prolonged therapeutic effects of Stattic. Herein, we propose that STAT3 inhibitor could effectively modulate specific macrophage populations in the L-AKI model and identify novel pathways of STAT3-dependent kidney macrophage trafficking.

## Methods

### L-AKI model

Male C57BL/6 mice (8 weeks old, *n* = 6–10 in each group) were purchased from KOATECH (South Korea) and intraperitoneally injected with LPS (10 mg/kg, cat. L2630, Sigma-Aldrich, St. Louis, MO, USA) for L-AKI model, which was reconstituted with distilled PBS. The mice, divided into four groups based on the time elapsed since LPS injection (0, 6, 12, and 24 h), were sacrificed corresponding to their respective time points post-injection. For the LPS + Stattic-treated group, the STAT3 inhibitor (5 mg/kg, 10 mg/kg) (Stattic; 6-nitrobenzo[b]thiophene-1,1-dixodide, cat. S7947, Sigma-Aldrich) was injected 1 h before LPS induction in the peritoneal region, and the mice were sacrificed 6 h after LPS injection. The control group consisted of vehicle-treated sham control mice. Blood samples were collected at sacrifice via an abdominal aortic puncture to evaluate renal function. All animal studies were performed under the guidance of the Institutional Animal Care and Use Committee (IACUC: 23–0055-S1A0) of Seoul National University Hospital.

### L-CKD model

In the L-CKD model, mice received intraperitoneal injections of LPS (1 mg/kg, Sigma-Aldrich) and Stattic (5 mg/kg, 10 mg/kg, Sigma-Aldrich) every 2 days, following a similar administration schedule as used in the L-AKI model. All animals were sacrificed after 14 days in the L-CKD model [14].

### Assessment of kidney function

Body weights and blood samples were obtained at 0, 6, 12, and 24 h after LPS injection. Blood urea nitrogen (BUN) (mg/dL) and creatinine (mg/dL) concentrations were measured by determining the rate of the modified Jaffe reaction using an autoanalyzer (HITACHI7180, Hitachi Chemical Industries) [22].

### Flow cytometry

Intrarenal mononuclear cells were isolated from mouse kidneys, and homogenates were obtained using a Stomacher H 80 Biomaster (Seward, Worthing, Sussex, UK). Single-cell suspensions were obtained by passing the tissue through a 40-μm cell strainer. Kidneys were resuspended in 40% Percoll (Amersham Pharmacia Biotech, Piscataway, NJ, USA) and overlaid on 80% Percoll. After being centrifuged for 30 min at 3000 rpm and 25 °C, renal mononuclear cells were isolated from the interface and washed in PBS [23]. Cells were diluted in 100 μL chilled FACS buffer (1X Hank’s Balanced Salt Solution; cat. 14025–092, Gibco, Billings, MT, USA) with 0.5% BSA and 0.05% sodium azide and incubated with mouse Fc block (cat. 5531–42, BD Biosciences, Franklin Lakes, NJ, USA) for 10 min. For surface marker staining, the cells were incubated with 1:100 dilutions of CD45-BV785 (cat. 103149; BioLegend, San Diego, CA, USA), CD11b-FITC (cat. 101206, BioLegend), and F4/80-PerCP Cy5.5 (cat. 157318; BioLegend) for 30 min at 25 °C. Finally, the cells were washed and resuspended in FACS buffer. For intracellular staining, the cells were stained with CD206-BV605 (cat. 141721, BioLegend) and phospho-STAT3 (Tyr 705)-APC (cat. 17–9033-42, Invitrogen, Waltham, MA, USA) using eBioscience™ IC Fixation Buffer (cat. 00–8222-49, Invitrogen) according to the manufacturer’s protocol. Stained cells were analyzed using a BD LSRFortessa™ device with BD FACSDiva™ software (BD Biosciences). Data were processed using FlowJo (v10.8.1., BD Biosciences).

### Protein purification and Western blot analysis

Kidney proteins from LPS-treated mice were isolated using RIPA buffer (cat. RC2002-050–00, Biosesang, Yongin, Korea; 150 mM NaCl; 100 mM Na_3_VO_4_; 50 mM Tris; HCL, pH 7.3; 0.1 mM EDTA 1% (vol/vol) sodium deoxycholate; 1% (vol/vol) Triton X-100; and 0.2% NaF) with protease inhibitor (GeneDEPOT, Katy, TX, USA). Kidney tissue lysates were obtained using steel beads in RIPA buffer with a TissueLyser instrument (Qiagen, Hilden, Germany) set at 30 strokes/s for 5 min. BCA assay was used to measure unknown protein concentrations. Protein lysates were electrophoresed in glycine-sodium dodecyl sulfate buffer and transferred onto a polyvinylidene difluoride membrane (Millipore, Bedford, MA, USA) on ice. The membrane was blocked for 1 h using blocking solution and probed with antibodies against pSTAT3 (cat. 9145L, Cell Signaling Technology, Danvers, MA, USA), STAT3 (cat. 9132L, Cell Signaling Technology), NGAL (cat. Sc-515876, Santa Cruz Biotechnology, Dallas, TX, USA), β-actin (cat. A1978, Sigma-Aldrich), ICAM-1 (cat. A00171, Boster Bio, Pleasanton, CA, USA), GAPDH (cat. 2118, Cell Signaling Technology), IL-6 (cat. CAB14687, Assay Genie, Dublin, Ireland), KIM-1 (cat. Ab233720, Abcam, Cambridge, UK), and Fibronectin (cat. Ab2413, Abcam). For the secondary antibodies, anti-rabbit IgG (cat. 7074S, Cell Signaling Technologies) and anti-mouse IgG (cat. 7076S, Cell Signaling Technologies) were used. Targeted proteins were detected using ImageQuant™ Las 4000 mini (GE HealthCare, Chicago, IL, USA) and analyzed using the ImageJ software (ImageJ v. 1.52; Wayne Rasband, National Institutes of Health).

### Immunohistochemistry (IHC) and histological analysis

The kidney tissue sections were fixed in 10% formalin and embedded in paraffin overnight. Then, 4 μm-thick sections of the kidneys were deparaffinized with xylene and rehydrated with ethanol. Briefly, the kidney sections were microwaved with sodium citrate buffer for antigen retrieval. Hydrogen peroxide (3%) was used to block endogenous peroxidase activity after dilution in methyl alcohol. The sections were probed with antibodies against F4/80 (cat. 70076 s, Cell Signaling Technology), ICAM-1 (cat. A00171, Boster Bio), NGAL (cat. Sc-515876, Santa Cruz Biotechnology), and pSTAT3 (cat. 9145L, Cell Signaling Technology). Dako Envision^+^ System-HRP-labeled polymer anti-rabbit (cat. K4003, Agilent DAKO, Santa Clara, CA, USA) and anti-mouse (cat. K4001, Agilent DAKO) antibodies were added and incubated for 1 h at 25 °C. Periodic Acid-Schiff (PAS) staining was conducted to evaluate the tubular injury scores based on the percentage of injured area, with cell nuclei counterstained using Mayer’s hematoxylin (Sigma-Aldrich) [24, 25]. The injury scores were determined as follows: 0 for no damage; 1 for an injured area of 1–10%; 2 for 11–25%; 3 for 26–75%; and 4 for an injured area of 75% or greater [26–29]. Sirius red (cat. Ab150681, Abcam) staining was used to evaluate the fibrotic changes in L-CKD kidney. For IHC, the stained area was examined and quantified using a Leica inverted microscope (Leica Camera, Wetzlar, Germany) and the LAS-4000 program (Leica Camera).

### RNA isolation and real-time qPCR

Total RNA was isolated from mouse kidney tissues using TRIzol reagent (Thermo Fischer Scientific, Waltham, MA, USA) following the manufacturer’s protocol. cDNA obtained from the total RNA of mouse kidney tissues was amplified using the ReverTra Ace qPCR RT Master Mix (Toyobo, Osaka, Japan). The gene expression levels were detected using EvaGreen qPCR Mastermix (Applied Biological Materials, Richmond, Canada), and real-time PCR was performed on the CFX Connect Real-Time PCR Detection System (Bio-Rad Laboratories, Hercules, CA, USA) with the following PCR conditions: 95 °C for 10 min, followed by 40 cycles of 95 °C for 10 s, 60 °C for 10 s, and 72 °C for 20 s. Relative quantification was conducted using the comparative CT (ΔΔCT) method, and each target gene expression level was normalized relative to GAPDH expression. The sequence of qPCR primers used is listed in Table 1.

**Table 1:** Primer sequences for real-time qPCR

Targets (Mouse)	Sense (5’ → 3’)	Antisense (5’ → 3’)
GAPDH	TATGTCGTGGAGTCTACTGGT	GAGTTGTCATATTTCTCGT
Ngal	ATGTCACCTCCATCCTGGTCAG	GCCACTTGCACATTGTAGCTCTG
Kim-1	AGGAACGAAATTTGCACATCAGT	CAAAGTGACGGCTCTGGTAGTCCT
Icam-1	AAACCAGACCCTGGAACTGCAC	GCCTGGCATTTCAGAGTCTGCT
IL-1β	TCGCTCAGGGTCACAAGAAA	CATCAGAGGCAAGGAGGAAAAC
Tnf-α	AGGGTCTGGGCCATAGAACT	CCACCACGCTCTTCTGTCAC
IL-22	GTGGGATCCCTGATGGCTGTCCTGCAG	AGCGAATTCTCGCTCAGACTGCAAGCAT
IL-23	TGAAAGAGACCCTACATCCCTTGA	CAGAAAATTGGAAGTTGGGATATGTT
p65	GCTGCCAAAGAAGGACACGACA	GGCAGGCTATTGCTCATCACAG
Cd11b	TCCGGATTCACTTCACCTTC	TTTTTGTCCTCCCATTCAGC
Mcp-1	GCTACAAGAGGATCACCAGCAG	GTCTGGACCCATTCCTTCTTGG
iNOS	ATGGTTGGTAACTTCCCCGT	TAAACCGACCAGGGAGGTCA
Cd206	GGAGCAGATGGAAGGTCTATGG	TGTCGTAGTCAGTGGTGGTTC
IL-6	ACCAGAGGAAATTTTCAATAGGC	TGATGCACTTGCAGAAAAACA
Jak2	GCTACCAGATGGAAACTGTGCG	GCCTCTGTAATGTTGGTGAGATC
Socs3	GGACCAAGAACCTACGCATCCA	CACCAGCTTGAGTACACAGTCG
Stat3	GCCACGTTGGTGTTTCATAATC	TTCGAAGGTTGTGCTGATAGAG
Akt3	GAGATGGATGCGTCTACAACCC	TCCACTTGCCTTCTCTCGAACC
Pik3r1	CAAACCACCCAAGCCCACTACT	CCATCAGCAGTGTCTCGGAGTT
Fn	TCCTGTCTACCTCACAGACTAC	GTCTACTCCACCGAACAACAA
Col1a1	CCTGGTAAAGATGGTGCC	CACCAGGTTCACCTTTCGCACC
IL-21	GCCTCCTGATTAGACTTCGTCAC	CAGGCAAAAGCTGCATGCTCAC

### Processing of sequencing data

Total RNA from the kidneys of L-AKI mice was sequenced and analyzed by EBIOGEN, Inc. (Seoul Korea). A TapeStation 4000 system (Agilent Technologies, Amstelveen, Netherlands) was used to assess RNA quality. Briefly, an RNA sequencing (RNA-seq) library was generated using a CORALL RNA-seq V2 Library Prep Kit (LEXOGEN GmbH, Vienna, Austria). mRNA was isolated using a Poly (A) RNA Selection Kit (LEXOGEN GmbH). To obtain high-throughput sequencing results, paired-end 100 bp sequencing was carried out using a NovaSeq 6000 (Illumina, San Diego, CA, USA). The Excel-based ExDEGA software from EBIOGEN was employed to identify differentially expressed genes (DEGs) and gene ontologies [30]. KEGG pathway enrichment analysis was performed using the enrichKEGG function, and GO enrichment analysis was conducted using the enrichGO function from the clusterprofiler package (v. 4.6.2) [31]. These analyses were used to identify significant pathways and GO profiles associated with DEGs.

### Cell culture

The murine macrophage cell line RAW264.7 (TIB-71) and the proximal tubule epithelial cell line OK (CRL-1840) were obtained from the American Type Culture Collection (ATCC, Manassas, VA, USA). RAW264.7 cells were cultured in Dulbecco’s Modified Eagle’s medium (DMEM, cat. L0103, BioWest, Nuaillé, France), and OK cells were cultured in Minimum Essential Medium Eagle (MEM, cat. M4655, Sigma-Aldrich). Both media were supplemented with 10% fetal bovine serum (FBS, cat. S1480, BioWest) and 1% penicillin/streptomycin (cat. 15,140–122, Gibco), and the cells were maintained at 37℃ with 5% CO_2_.

### Establishment of an in vitro model for LPS-induced inflammation

To evaluate the effect of LPS-induced inflammation, RAW264.7 cells were treated with LPS (cat. L2630, Sigma-Aldrich) in a time-dependent (0.5 μg/mL LPS for 6, 12, and 24 h) or dose-dependent manner (0.25 μg/mL and 0.5 μg/mL). For the STAT3 inhibition, Stattic (1 μM or 2 μM, cat. S7947, Sigma-Aldrich) was administered to the cells 1 h before LPS induction. The expression of pSTAT3 and iNOS in RAW264.7 cells were then observed using FACS analysis.

### Co-culture system of macrophages and tubular epithelial cells

RAW264.7 cells and OK cells were pretreated with Stattic (1, 2 μM) 1 h before being exposed to 0.5 μg/mL LPS for 12 h. After incubation, the cells were collected for further experiments to observe the expression of IL-6, KIM-1, pSTAT3, and STAT3. For the co-culture system, OK cells (5 × 10^5^) were plated and cultured in MEM media a day before co-culture. Furthermore, to confirm macrophage-driven inflammation, RAW264.7 cells (1.5 × 10^6^), stimulated with or without LPS for 12 h were then added to the OK cells. OK cells were treated with Stattic (2 μM) either 1 h before or at the start of co-culture to evaluate the effects of pre/concomitant-treatment. After 12 h of co-culture, RAW264.7 cells were isolated with the MagniSort™ mouse CD11b positive selection kit (cat. 8802–6860-74, Thermo Fisher Scientific) following the manufacturer’s protocol. Subsequently, OK cell lysates were analyzed by western blot to assess the protein levels of KIM-1.

### Statistical analysis 

All results are expressed as the mean ± standard error of the mean (SEM). Statistical analyses were performed using an unpaired two-tailed Student’s t-test in GraphPad Prism 9.0 (GraphPad Software Inc., San Diego, CA, USA). In KEGG and GO enrichment analyses, the *p*-values were calculated using the R packages enrichGO and enrichKEGG. Each experiment was independently repeated three times. A *p-value* less than 0.05 was considered statistically significant (*P < 0.05, **P < 0.01, ***P < 0.001).

## Results

### LPS injection leads to AKI and alters F4/80^+^ CD11b^+^ cell subpopulation over time

**Fig. 1:**
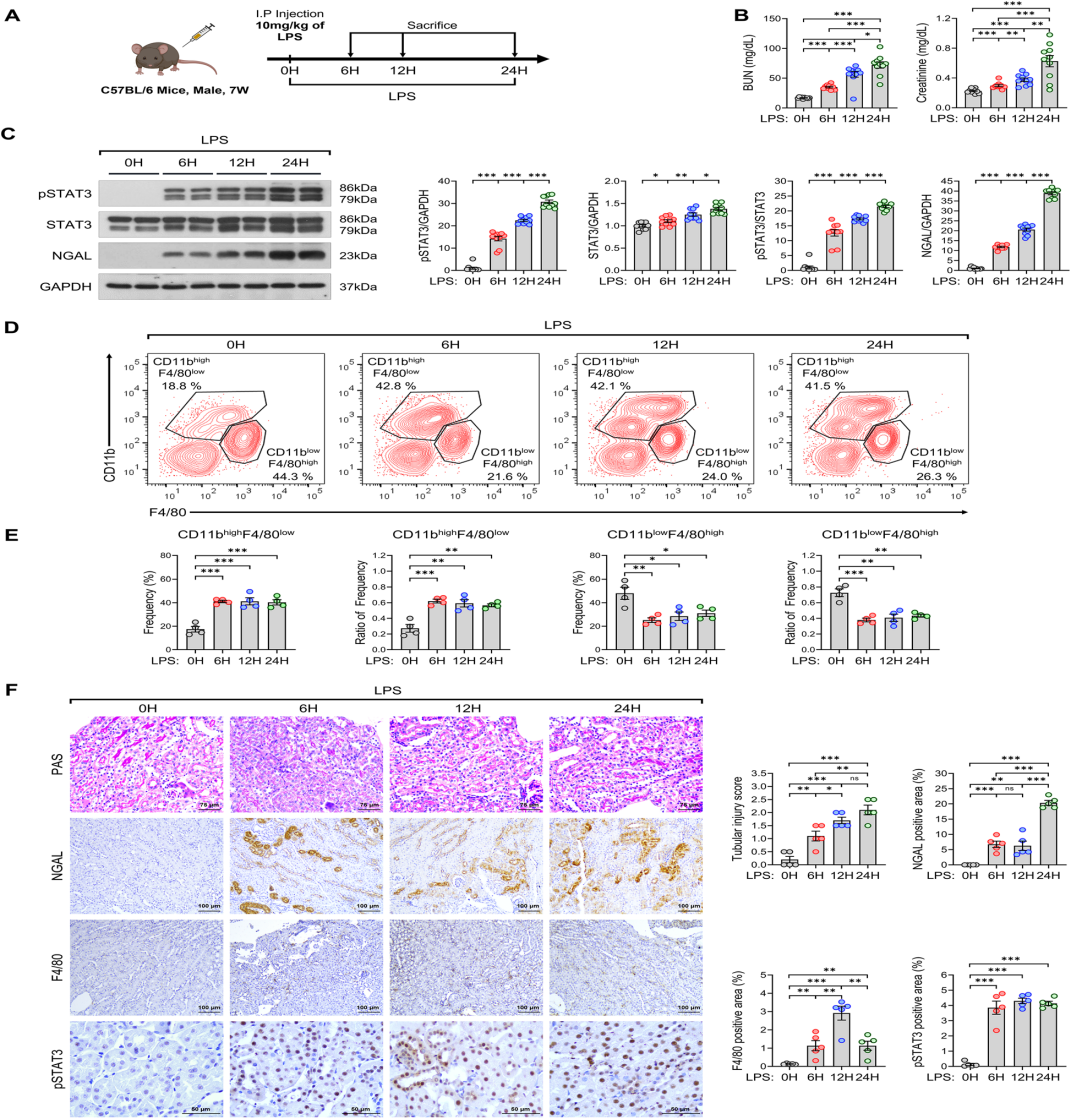
Time-dependent effect of LPS on macrophage subpopulations co-expressed with F4/80 and CD11b. **A** Schematic diagram of the progression of L-AKI. **B** Kidney functions were evaluated at various time points following LPS injection in mice: 0, 6, 12, and 24 h (*n* = 10 in each group). **C** Western blot analysis of the expression of pSTAT3, STAT3, and NGAL after LPS injection. **D** and **E** Flow cytometry results of immune cell types in the mouse L-AKI model, representing two populations (kidney macrophages, CD11b^high^F4/80^low^ and CD11b^low^F4/80^high^) (**D**); the frequency and ratio of individual populations were quantified using GraphPad Prism (*n* = 4 in each group) (**E**). **F** IHC representative images (left) and quantification (right) of NGAL (*n* = 5 in each group), F4/80 (*n* = 5 in each group), pSTAT3 (*n* = 5 in each group), and PAS from LPS-treated mice. Scale bars, 75 μm (200X), 100 μm (100X), and 50 μm (400X). All experiments were independently replicated at least three times, and the data are presented as mean ± SEM. *P < 0.05, **P < 0.01, ***P < 0.001

L-AKI manifests as immune cell infiltration and inflammation, leading to acute renal dysfunction. To determine the optimal timeframe for LPS injection, we tested three distinct time intervals and subsequently assessed renal function in each scenario (Fig. 1A). Following LPS treatment, BUN and creatinine levels increased across different courses, indicating that kidney function deteriorated over time (BUN: 16.91 ± 0.41, 35.05 ± 1.25, ***P < 0.001; 56.57 ± 4.90, ***P < 0.001; 70.89 ± 6.61, ***P < 0.001; creatinine: 0.23 ± 0.01, 0.30 ± 0.01, ***P < 0.001; 0.38 ± 0.02, ***P < 0.001; 0.62 ± 0.10, ***P < 0.001; 0 h *vs* 6, 12, and 24 h). Notably, mice treated with LPS for 6 h exhibited a rapid increase compared with the control group (Fig. 1B). The expression changes of kidney injury marker NGAL accelerated considerably 6 h after LPS injection, peaked at 12 h, and remained elevated until 24 h. Additionally, pSTAT3 expression was rapidly upregulated at 6 h of LPS induction (Fig. 1C). In L-AKI time-dependent injection mice, the mRNA expression of kidney injury markers, immune response, pro-inflammatory related, and STAT3-related genes was considerably increased by LPS stimulation. However, CD206 mRNA expression was significantly decreased (Figure S1C). These results indicate that inflammatory responses to LPS were accelerated 6 h after injection, and persisted until 24 h. Based on these findings, we hypothesized that early-stage inhibition is effective and that this timeframe would correlate with an increase in immune cell levels. According to findings reported in multiple studies, it can be inferred that the CD11b^high^F4/80^low^ population represents infiltrating macrophages in the kidney, while the CD11b^low^F4/80^high^ population is indicative of kidney resident macrophages [9, 32–35]. We next determined the changes that occurred in these two macrophage subpopulations over time after LPS injection. Our FACS analysis showed a marked shift in immune cell populations: the CD11b^high^F4/80^low^ population increased from 17.5% ± 2.4% to 40.3% ± 2.2% (***P < 0.001) after 24 h, whereas the CD11b^low^F4/80^high^ population declined from 48% ± 5.2% to 31% ± 2.7% (*P < 0.05) within the same period. Similarly, when comparing the ratio of each macrophage population, we found that CD11b^high^F4/80^low^ population increased, while CD11b^low^F4/80^high^ population decreased after LPS injection (Fig. 1D, E). L-AKI was detected in the kidney cortex tubules of mice subjected to LPS challenge, exhibiting symptoms such as dilation of the renal capsule cavity, edema in renal tubular epithelial cells, collapse of epithelial cells due to local focal necrosis, and loss of tubular brush borders. Consistent with our earlier observations, the infiltration of F4/80-positive immune cells and the expression of NGAL and pSTAT3 progressively increased following LPS injection (Fig. 1F, Figure S1A). Considering a comprehensive analysis of the preceding results, we found that the most pronounced changes occurred 6 h after LPS injection, suggesting that this is the ideal time for AKI induction. Together, these findings indicate that the main role of a specific macrophage subpopulation in the kidney is either to facilitate recovery or potentially exacerbate L-AKI.

### STAT3 inhibitor ameliorates L-AKI

**Fig. 2:**
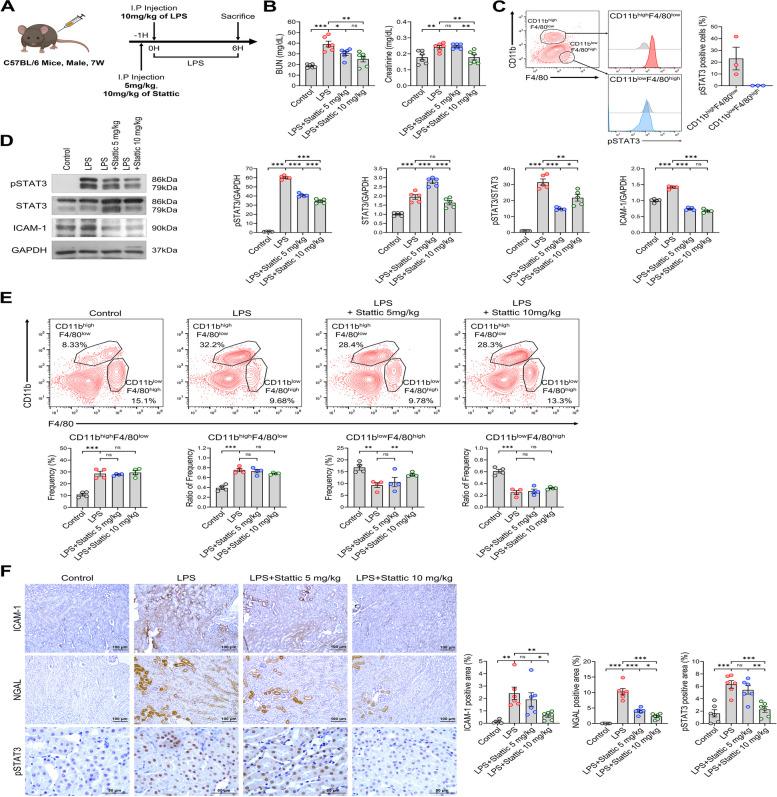
STAT3 inhibitor attenuates L-AKI and restores kidney function. **A** Schematic diagram of the animal model. **B** BUN and serum creatinine were measured to assess kidney function. Groups: Control, LPS, LPS + Stattic (5 mg/kg) and LPS + Stattic (10 mg/kg) (*n* = 6 in each group). **C** Flow cytometric comparison of pSTAT3 levels was conducted between two populations (kidney macrophages, CD11b^high^F4/80^low^ and CD11b^low^F4/80^high^) in LPS-induced kidneys. **D** Western blotting representative image (left) and quantification (right) of pSTAT3, STAT3 and ICAM-1. Each band shows a typical group, as indicated. **E** Changes in the proportion of two macrophage subpopulations between each group were observed with FACS analysis, and the frequency and ratio of individual populations were quantified using GraphPad Prism. **F** Upregulated expression of ICAM-1, NGAL, and pSTAT3 by LPS induction in IHC analysis (*n* = 6 in each group) decreased with Stattic treatment. Scale bars, 100 μm (100X) and 50 μm (400X). All experiments were independently replicated at least three times, and the data are presented as mean ± SEM. *P < 0.05, **P < 0.01, ***P < 0.001

Following inflammation, STAT3 is ubiquitously expressed, and many studies have highlighted the protective role of STAT3 inhibitors in mitigating AKI damage. To examine the potential therapeutic effect of a STAT3 inhibitor (Stattic) in an L-AKI mouse model, we administered Stattic (5 mg/kg; low dose or 10 mg/kg; high dose) 1 h before LPS injection (Fig. 2A). In L-AKI mice, low dose Stattic treatment reduced BUN level, but a more pronounced effect was observed with the high dose, significantly reducing both BUN and blood creatinine levels to 25.10 ± 2.66 and 0.18 ± 0.02 (**P < 0.01) (Fig. 2B). Considering that STAT3 plays a crucial role in the association between kidney disease and macrophages, we elucidated the effects of STAT3 inhibitor in an L-AKI model. As supporting evidence, we analyzed pSTAT3 levels in CD11b^high^F4/80^low^ and CD11b^low^F4/80^high^ macrophage populations using FACS, which was dynamically switched by LPS injection. The percentage of pSTAT3-positive cells in the CD11b^high^F4/80^low^ population was higher than in the CD11b^low^F4/80^high^ macrophage population following LPS injection (Fig. 2C). Based on these results, we anticipated that Stattic treatment in the L-AKI model would influence macrophage phenotypes. Consistent with these functional observations, Stattic, when administered 1 h before LPS exposure, significantly decreased the protein levels of the inflammatory markers ICAM-1 and pSTAT3 (Fig. 2D). In L-AKI mice treated with Stattic, an increase in total STAT3 was observed. However, the level of the active form of STAT3, phospho-STAT3 (Y705), was markedly decreased in mice treated with Stattic. We observed that Stattic treatment restored the frequency of CD11b^low^F4/80^high^ to that before LPS injection, which was reduced by L-AKI. The frequency of the CD11b^low^F4/80^high^ population, which decreased from 16.8% ± 1.118% to 9.3% ± 1.027% (**P < 0.01) by LPS, was restored to 13.7% ± 0.433% (**P < 0.01) with Stattic (10 mg/kg) treatment. In addition, the ratio of the CD11b^low^F4/80^high^ population, which decreased from 0.61 ± 0.032 to 0.25 ± 0.032 (***P < 0.001) by LPS, was restored to 0.32 ± 0.01 (ns; *P* = 0.0786) with Stattic (10 mg/kg) treatment. On the other hand, the ratio of the CD11b^high^F4/80^low^ population increased from 0.39 ± 0.032 to 0.75 ± 0.032 (***P < 0.001) by LPS but slightly decreased to 0.68 ± 0.01 (ns; *P* = 0.0786) with Stattic (10 mg/kg) treatment (Fig. 2E). Although we also examined the changes in macrophage populations with Stattic treatment at 5 and 10 mg/kg, there were no significant changes in macrophage populations with Stattic 5 mg/kg treatment. However, we observed a notable restoration of the CD11b^low^F4/80^high^ population with Stattic 10 mg/kg treatment. Histological analysis revealed that the STAT3 inhibitor considerably decreased the expression of ICAM-1, NGAL, and pSTAT3 in the L-AKI mouse model in a dose-dependent manner. (Fig. 2F, Figure S1B). The group treated with LPS also exhibited increased STAT3 phosphorylation and translocation into the nucleus within kidney tubular cells. Stattic treatment alleviated this effect. These results showed that STAT3 inhibition effectively mitigated inflammation by retrieving the CD11b^low^F4/80^high^ population, suggesting the therapeutic potential of STAT3 inhibitor in alleviating kidney injury in the L-AKI model.

### Transcriptome profile measurement with STAT3 inhibitor

**Fig. 3:**
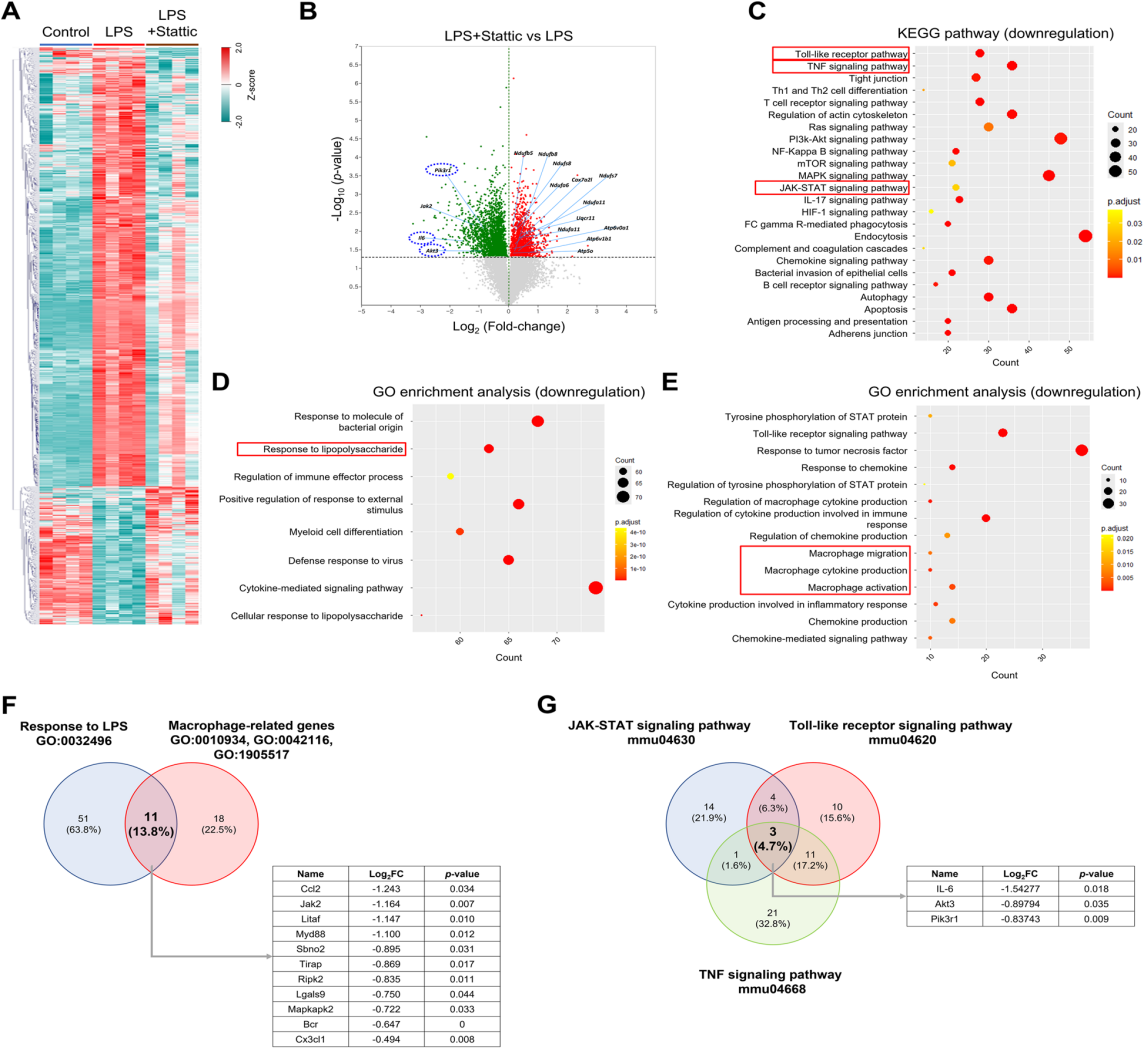

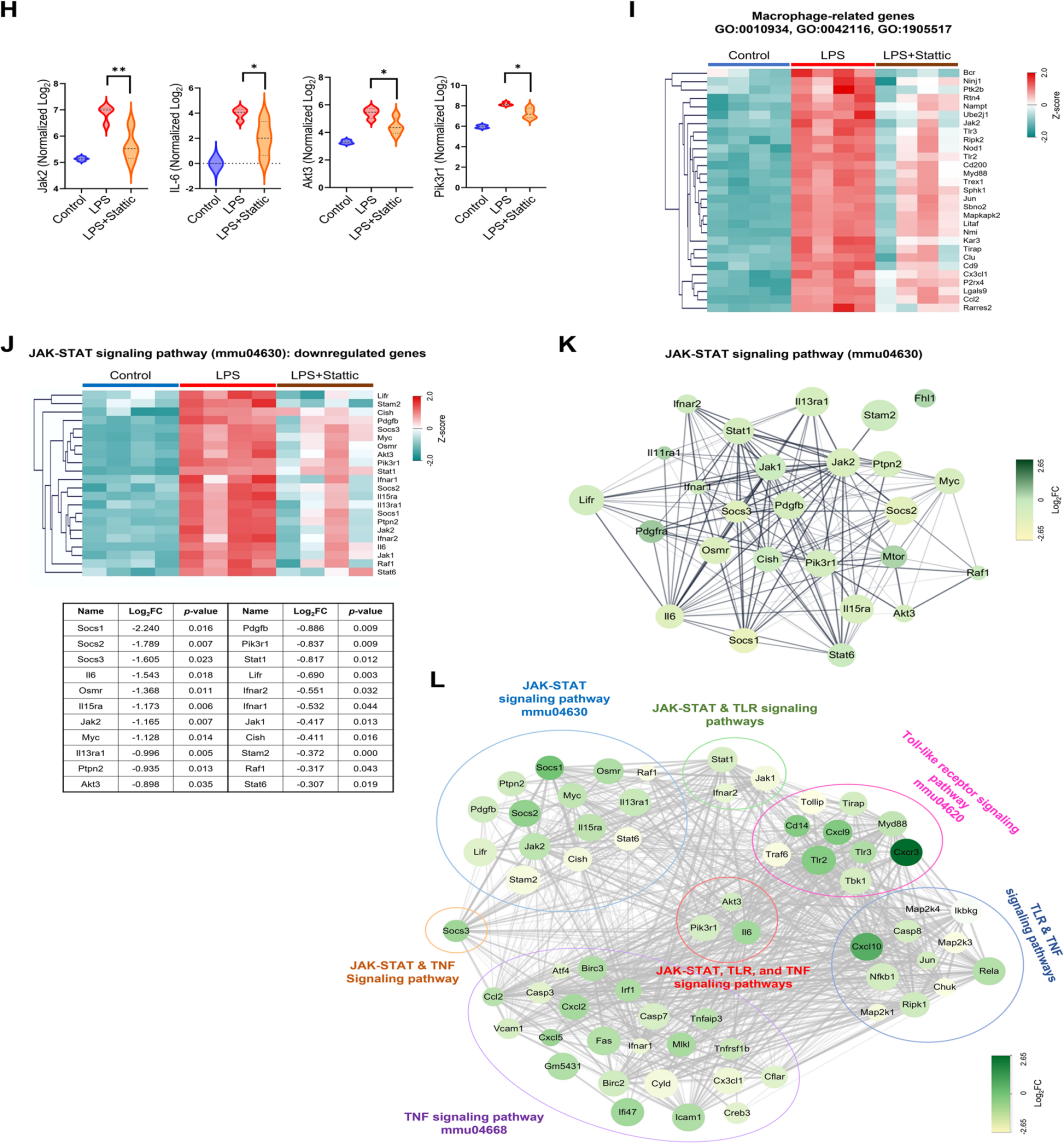
Stattic alters immune-related gene expression in L-AKI mice. **A** Heatmap showing DEGs in the control, LPS, and LPS + Stattic-treated group (*n* = 4 in each group). **B** Volcano map showing DEGs involved in KEGG pathways and GO terms. Green and red dots represent downregulated and upregulated genes, respectively. **C**, **D**, **E** The dotted plot presents the KEGG and GO enrichment analyses of the 1,414 downregulated genes. **F**, **G** The intersection of genes identified by GO terms (**F**) and KEGG pathways (**G**) is shown. 11 genes were associated with the response to LPS (GO:0032496) and macrophage-related genes (GO:0010934, GO:0042116, GO:1905517), and 3 genes were common to the JAK-STAT (mmu04630), TLR (Toll-like receptor, mmu04620), and TNF (Tumor necrosis factor, mmu04668) signaling pathways. **H** Violin plots depicting the expression levels of the intersecting genes from (**F**, **G**), with mean ± SEM. *P < 0.05, **P < 0.01, ***P < 0.001. **I** The DEGs related to macrophage function were categorized using the following GO terms: macrophage cytokine production (GO:0010934), macrophage activation (GO:0042116), and macrophage migration (GO:1905517). **J** A representative heatmap of the JAK-STAT signaling pathway (mmu04630) showing downregulated genes is presented. **K** Network analysis of downregulated and upregulated genes in the JAK-STAT signaling pathway was constructed, visualizing their interconnections. **L** Network analysis of host immune responses following LPS induction was demonstrated. JAK-STAT, TLR and TNF signaling pathways. The results are presented as mean ± SEM, and statistical analysis was conducted using an unpaired two-tailed Student’s t-test. *P < 0.05, **P < 0.01, ***P < 0.001

To investigate the immune profile in the mouse L-AKI model, we conducted mRNA sequencing analysis, identifying differentially expressed genes (DEGs) in the sham, LPS, and LPS + Stattic groups. Our findings showed that 444 genes were upregulated (FC > 1, log_2_ = 4, *P* < 0.050) and 1,414 genes were downregulated (FC < 1, log_2_ = 4, *P* < 0.050) following Stattic treatment in the L-AKI model (Fig. 3A, B). Top 10 KEGG and GO enrichment analyses of these DEGs revealed that Stattic influences macrophage migration, with an increase in various metabolic processes and a decrease in immune response upon pathogen recognition (Figure S2A, B). Network analysis confirmed that the JAK-STAT signaling pathway, along with other inflammatory pathways, was mediated by 3 key genes (Fig. 3L). LPS + Stattic treatment downregulated the JAK-STAT (mmu04630), TNF (mmu04668), and TLR (mmu04620) signaling pathways (Fig. 3C, Figure S3A). 3 common genes, *IL-6*, *Akt3*, and *Pik3r1*, part of the PI3K-AKT signaling pathway, were identified. This pathway is activated by IL-6/IL-6R (gp130) binding and influences cytokine expression in sepsis (Figure S4). These results support our hypothesis that Stattic inhibits the JAK-STAT signaling pathway through alterations in the PI3K-AKT signaling cascade [36].

**Fig. 4:**
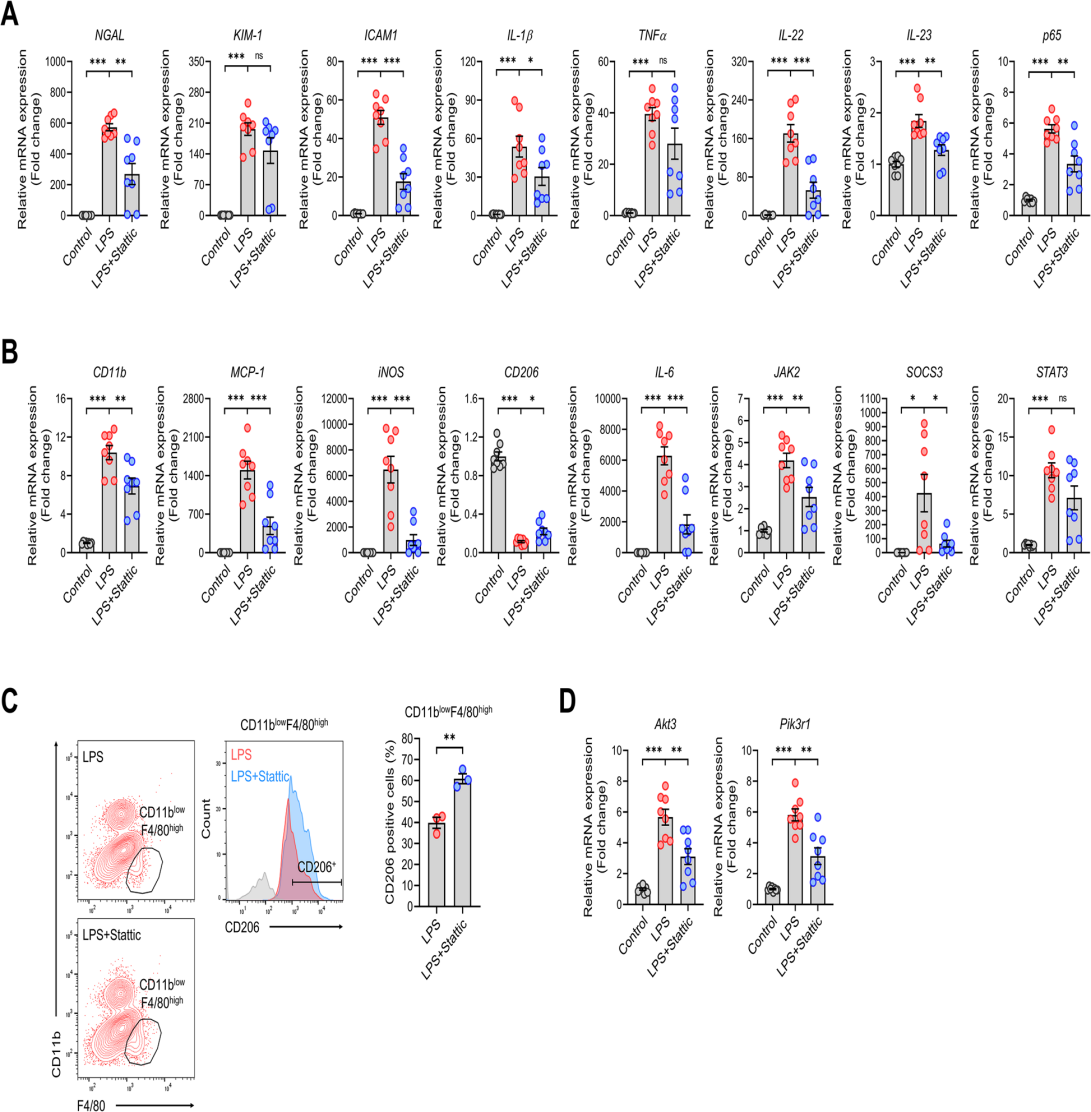
Kidney mRNA expression analysis of L-AKI mice by real-time qPCR. **A**, **B** The mRNA expression profile in the kidneys of L-AKI mice was investigated through real-time qPCR analysis (*n* = 8 in each group). Genes related to (**A**) kidney injury and proinflammation as well as (**B**) macrophages and the STAT3 pathway were analyzed 6 h after LPS injection with Stattic treatment. **C** The percentage of CD206-positive cells within the CD11b^low^F4/80^high^ population was compared between the LPS and LPS + Stattic groups with FACS analysis. **D** The mRNA levels of two genes commonly associated with the JAK-STAT, TLR, and TNF pathways were analyzed using real-time qPCR (*n* = 8 in each group). The results are presented as mean ± SEM, and statistical analysis was conducted using an unpaired two-tailed Student’s t-test. *P < 0.05, **P < 0.01, ***P < 0.001

GO enrichment analysis showed three macrophage-specific terms significantly regulated by LPS: macrophage-related genes (GO:0010934, GO:0042116, GO:1905517) and response to LPS (GO:0032496) (Fig. 3D, E, I, Figure S3B). 11 common genes were identified, with *Jak2* and *Myd88* central in the network analysis (Figs. 3F, Figure S7). Other altered genes, including *MCP-1 (Ccl2)* and *Cx3c11*, are involved in cytokine secretion or macrophage activation (Figure S5). Further analysis of downstream JAK-STAT pathway genes under LPS and Stattic exposure revealed 22 downregulated genes (Fig. 3J) and 4 upregulated genes (Figure S6A). Network and KEGG analyses highlighted comprehensive interactions involving the PI3K-Akt and JAK-STAT pathways (Fig. 3K, Figure S6B). Based on our previous KEGG result, the *IL-6* expression was decreased after Stattic treatment. Here, we employed the IL-6-mediated STAT3 pathway to observe a potential mechanism of Stattic in L-AKI condition. Notably, 11 genes (Figure S6C) were identified as downstream genes of STAT3 signaling pathway in the context of canonical and noncanonical pathway (Figure S6D). The cross-talk of STAT3 signaling pathway related genes was also observed (Figure S6E). Overall, strong downregulation in LPS + Stattic-treated mice suggests potential therapeutic targets in the L-AKI mouse model.

### STAT3 inhibitor treatment reduces the expression of L-AKI-associated transcriptome in kidneys

We conducted real-time qPCR analysis of kidney mRNA in a mouse model of L-AKI to confirm the transcriptome profile using RNA-seq. The kidney injury marker genes (*NGAL* and *KIM-1*) and proinflammatory genes (*ICAM-1*, *IL-1β*, *TNF-α*, *IL-22*, *IL-23*, and *p65*) were increased in kidney tissues from L-AKI mice. However, Stattic treatment attenuated the expression of these genes (Fig. 4A). In addition, the expression of macrophage-related genes (*CD11b*, *MCP-1*, *iNOS,* and *CD206*) and STAT3 pathway genes (*STAT3*, *IL-6*, *JAK2*, and *SOCS3*) was analyzed. We found that the expression of genes involved in macrophage phenotypes, such as *CD11b*, *MCP-1,* and *iNOS,* was induced by LPS stimulation and alleviated by Stattic treatment, along with the expression of STAT3-associated genes, such as *STAT3*, *IL-6*, *JAK2*, and *SOCS3*. The LPS-induced elevation in the mRNA levels of *MCP-1* suggests that the substantial increase in the CD11b^high^F4/80^low^ population in the kidney may be attributed to the increased expression of chemoattractants, which recruit macrophages. In contrast, M2-related *CD206* expression showed conflicting expression patterns compared to other LPS-induced genes (Fig. 4B). Based on the qPCR results for the mRNA levels of *CD206*, we analyzed the changes in the CD206 macrophage phenotype between the LPS and LPS + Stattic groups using FACS. Interestingly, the percentage of CD206-positive cells markedly increased in the CD11b^low^F4/80^high^ population after Stattic injection (Fig. 4C). In the RNA-seq analysis shown in Fig. 3, among the LPS-induced genes downregulated by Stattic treatment, *Akt3* and *Pik3r1* were identified as genes corresponding to the JAK-STAT, TLR, and TNF pathways. This phenomenon was confirmed using real-time qPCR (Fig. 4D). Intraperitoneal LPS administration affects the spleen, a secondary immune organ, before the kidneys. Therefore, we examined the mRNA expression in the spleens of L-AKI mice using real-time PCR to determine whether the transcriptome of the kidney tissue was reflected systemically. We also analyzed the expression of *IL-21*, which represents the proinflammatory signature in the spleen. The increased mRNA expression of proinflammatory and STAT3-associated genes induced by LPS stimulation was inhibited by Stattic treatment (Figure S8A, B). Overall, the real-time qPCR results for the kidneys and spleens in the L-AKI mice model showed a similar pattern to the RNA-seq results.

### STAT3 inhibitor alleviates LPS-induced inflammation in RAW264.7 cells

**Fig. 5:**
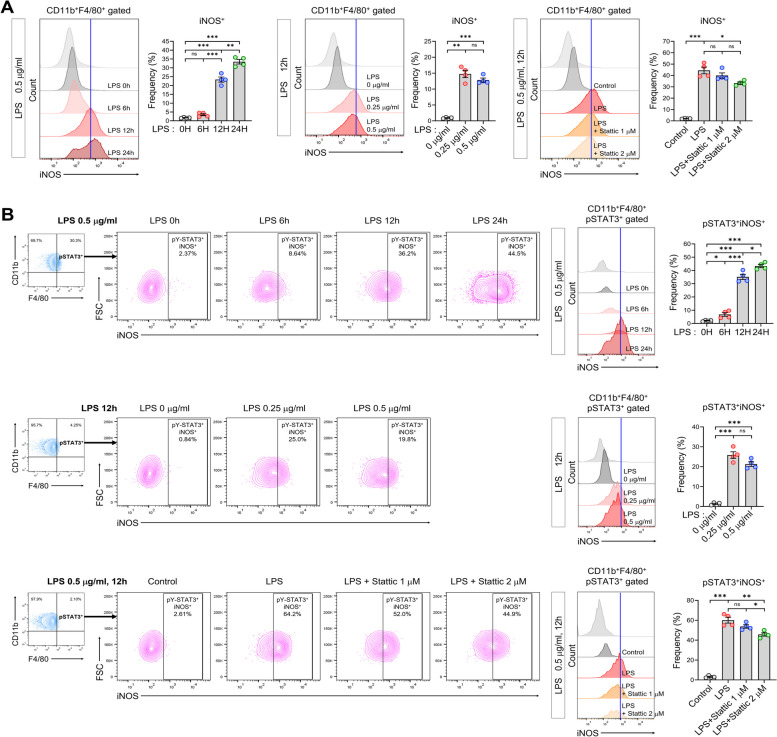
The inflammatory response induced by LPS stimulation in RAW264.7 cells. **A** Histogram and quantification graph of iNOS-positive cells using FACS analysis after time-dependent, dose-dependent LPS treatment, and Stattic treatment with LPS stimulation in RAW264.7 cells. **B** Histogram and quantification graph of pSTAT3^+^iNOS^+^ cells using FACS analysis after time-dependent, dose-dependent LPS treatment, and Stattic treatment with LPS stimulation in RAW264.7 cells. The results are presented as mean ± SEM, and statistical analysis was conducted using an unpaired two-tailed Student’s t-test. *P < 0.05, **P < 0.01, ***P < 0.001

We have observed changes in the L-AKI mouse model, so far. Next, we attempted to examine the effects of STAT3 inhibition on macrophages in LPS stimulation in vitro using FACS analysis. iNOS expression considerably increased 12 h after LPS treatment. This suggests that LPS induced inflammation in resting macrophages. Although we were unable to observe the dose-dependent effects of LPS, we confirmed that treatment with 0.25 µg/mL LPS alone considerably increased iNOS expression in RAW264.7 cells. LPS-induced iNOS expression was inhibited by treatment with 2 µM Stattic (Fig. 5A). We also observed the activation of STAT3 by LPS treatment and the inhibitory effect of Stattic on STAT3 (Figure S9). Furthermore, FACS analysis showed that inflammation in STAT3-activated macrophages was assessed, with a marked increase in the pSTAT3^+^iNOS^+^ cell population observed from 12 h after LPS treatment, even at a dose of 0.25 µg/mL. Moreover, treatment with 2 µM Stattic inhibited the pSTAT3^+^iNOS^+^ cell population, demonstrating a more extensive inhibitory effect compared to 1 µM Stattic (Fig. 5B). We believe that anti-inflammatory effects of STAT3 inhibition in LPS-stimulated RAW264.7 cells allowed us to understand the macrophage-specific changes in our L-AKI mice.

### Inhibition of STAT3 attenuates injury of tubular epithelial cells in co-culture system with macrophage

**Fig. 6:**
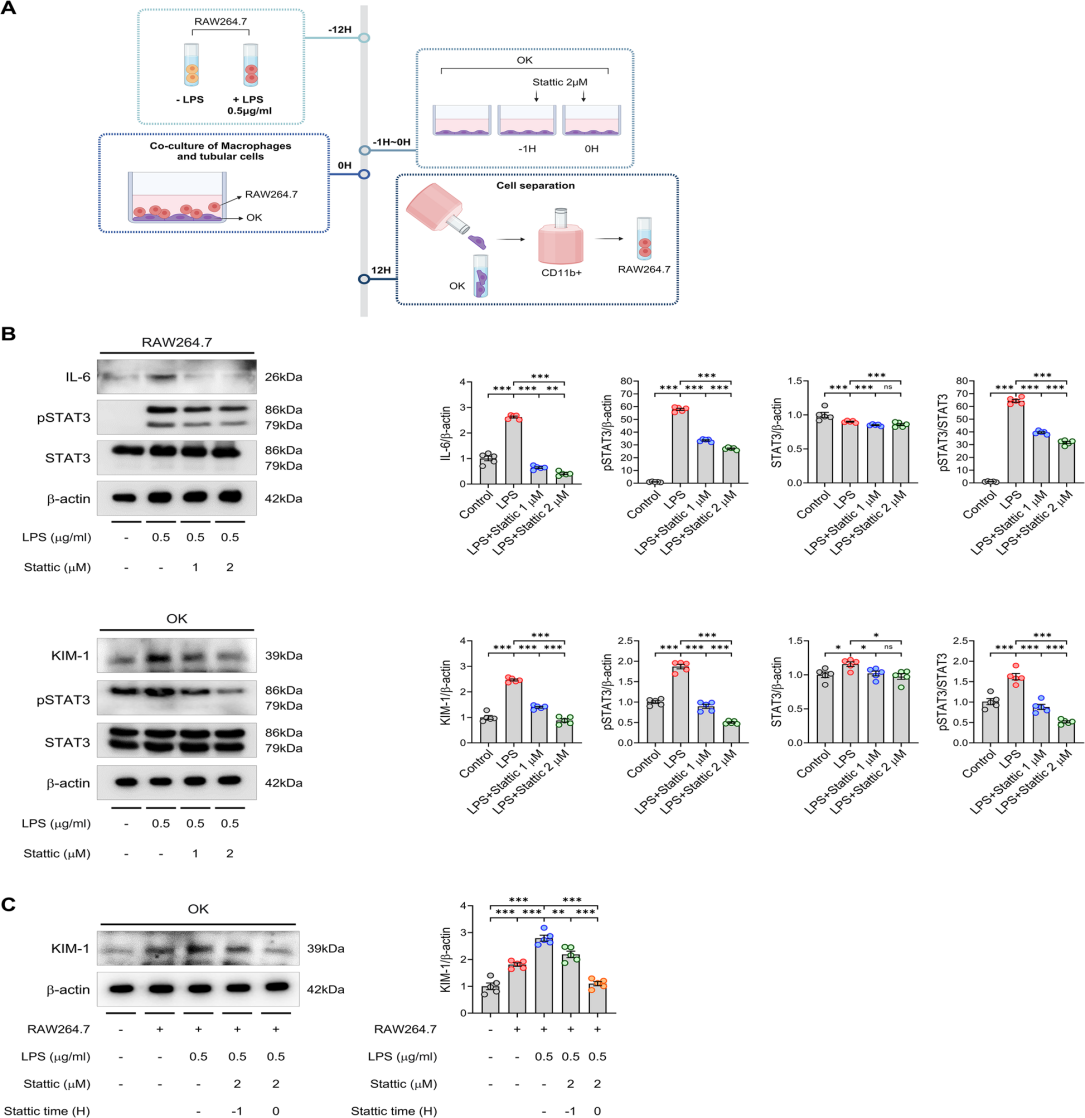
Inhibition of STAT3 attenuates tubular epithelial cell injury in a co-culture system with macrophages. **A** A schematic illustration of co-culture system with macrophages and tubular epithelial cells. **B** Western blot results of IL-6, KIM-1, pSTAT3, and STAT3 expression in RAW264.7 and OK cells are shown. **C** Upregulated expression of KIM-1 in OK cells co-cultured with LPS-stimulated RAW264.7 cells significantly decreased upon pre- and concomitant-treatment of 2 μM Stattic. All experiments were independently replicated at least three times, and the data are presented as mean ± SEM. *P < 0.05, **P < 0.01, ***P < 0.001

To examine the crosstalk between LPS-stimulated macrophages and tubular epithelial cells, we co-cultured OK cells with RAW264.7 cells (Fig. 6A). Initially, we assessed the effects of STAT3 inhibition on LPS-induced activation in each cell type independently (Fig. 6B). STAT3 inhibition with 1 and 2 µM Stattic markedly reduced LPS-induced pSTAT3 expression in both cell types. Additionally, LPS stimulation elevated IL-6 levels in RAW264.7 cells, which decreased following Stattic treatment, indicating an anti-inflammatory effect in macrophages. In OK cells, Stattic treatment also reduced KIM-1 levels, suggesting attenuation of LPS-induced tubular injury. OK cells co-cultured with LPS-stimulated RAW264.7 cells exhibited considerably higher levels of KIM-1 compared to those co-cultured with unstimulated RAW264.7 cells or cultured alone (Fig. 6C). The upregulation of KIM-1 was markedly reduced by the pre- and concomitant-treatment with 2 µM Stattic. These results underscore the role of STAT3 in macrophage-driven inflammation and its contribution to tubular epithelial cell injury in L-AKI, further supporting the potential of STAT3 inhibition as a therapeutic strategy.

## Anti-fibrotic effects of the STAT3 inhibitor in L-CKD mice

**Fig. 7:**
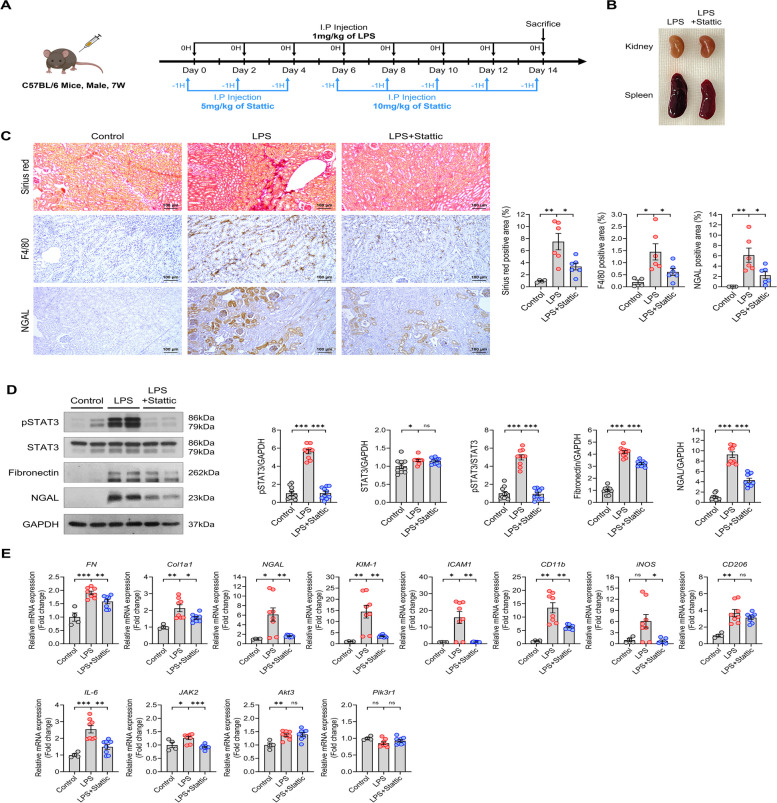
STAT3 inhibitor exhibits anti-fibrotic effects in L-CKD. **A** Schematic diagram of LPS and Stattic administration in L-CKD. **B** The gross morphology of kidney and spleen was compared between the LPS and LPS + Stattic group. **C** Sirius red staining, IHC representative images of F4/80 and NGAL (left), and quantification (right) of stained area (*n* = 6 in each group). Scale bars, 100 µm (100X). **D** Western blotting representative image (left) and quantification (right) of pSTAT3, STAT3, fibronectin, and NGAL. **E** The relative mRNA expression of fibrosis, immune responses, and STAT3-related genes. All experiments were independently replicated at least three times, and the data are presented as mean ± SEM. *P < 0.05, **P < 0.01, ***P < 0.001

We aimed to observe not only the injury-ameliorating effects of STAT3 blockade in L-AKI but also to investigate the effects of STAT3 inhibition in chronic kidney disease conditions. In the L-CKD in vivo experiment, LPS and Stattic injections were administered for 14 days (Fig. 7A). Interestingly, when the mice were sacrificed, we observed that the group injected with Stattic alongside LPS had smaller spleens compared to the group injected with LPS (Fig. 7B). This was considered the evidence that the STAT3 inhibitor may have reduced inflammation in L-CKD. Sirius red staining was conducted to evaluate the extent of fibrosis. The Sirius red-positive area, indicative of collagen deposition and interstitial fibrosis, was elevated in the LPS group but reduced by Stattic treatment. Furthermore, STAT3 inhibition decreased the expression of F4/80 and NGAL induced by LPS (Fig. 7C). Additionally, pSTAT3 expression in the kidneys of L-CKD mice was increased compared to that in control mice, and this was markedly reduced by Stattic treatment. Moreover, the expression of fibronectin was elevated in L-CKD, suggesting induced kidney fibrosis. However, with STAT3 inhibition, we observed a reduction in fibronectin expression. Similar results were also confirmed through the expression pattern of the kidney injury marker NGAL (Fig. 7D). Transcriptomic analysis further detailed the mRNA expression of L-CKD-related genes (Fig. 7E). The mRNA expression of fibrosis-related genes such as *FN* and *Col1a1* was enhanced by LPS injection but was alleviated by Stattic treatment. The mRNA expression of typical kidney injury markers like *NGAL* and *KIM-1*, as well as adhesion molecules and surface markers of immune cells like *ICAM-1* and *CD11b*, showed similar patterns. However, unlike in L-AKI, the expression of *CD206*, an M2-type macrophage marker, increased with LPS stimulation and was subsequently inhibited by Stattic. This different expression of *CD206* compared to L-AKI highlights a clear distinction between acute and chronic kidney injury, which we were able to further explore in related studies. In addition to genes related to kidney disease and immune response, we investigated the mRNA expression of STAT3 downstream genes. *IL-6* and *JAK2* showed the decreased expression by Stattic treatment in L-CKD, while *Akt3* and *Pik3r1* did not exhibit significant alleviated effects by STAT3 blockade. This suggests that the PI3K-AKT pathway mediated by STAT3 inhibition is specific to acute kidney injury. Ultimately, the L-CKD experiments demonstrate the anti-fibrotic effects of the STAT3 inhibitor.

## Discussion

**Fig. 8:**
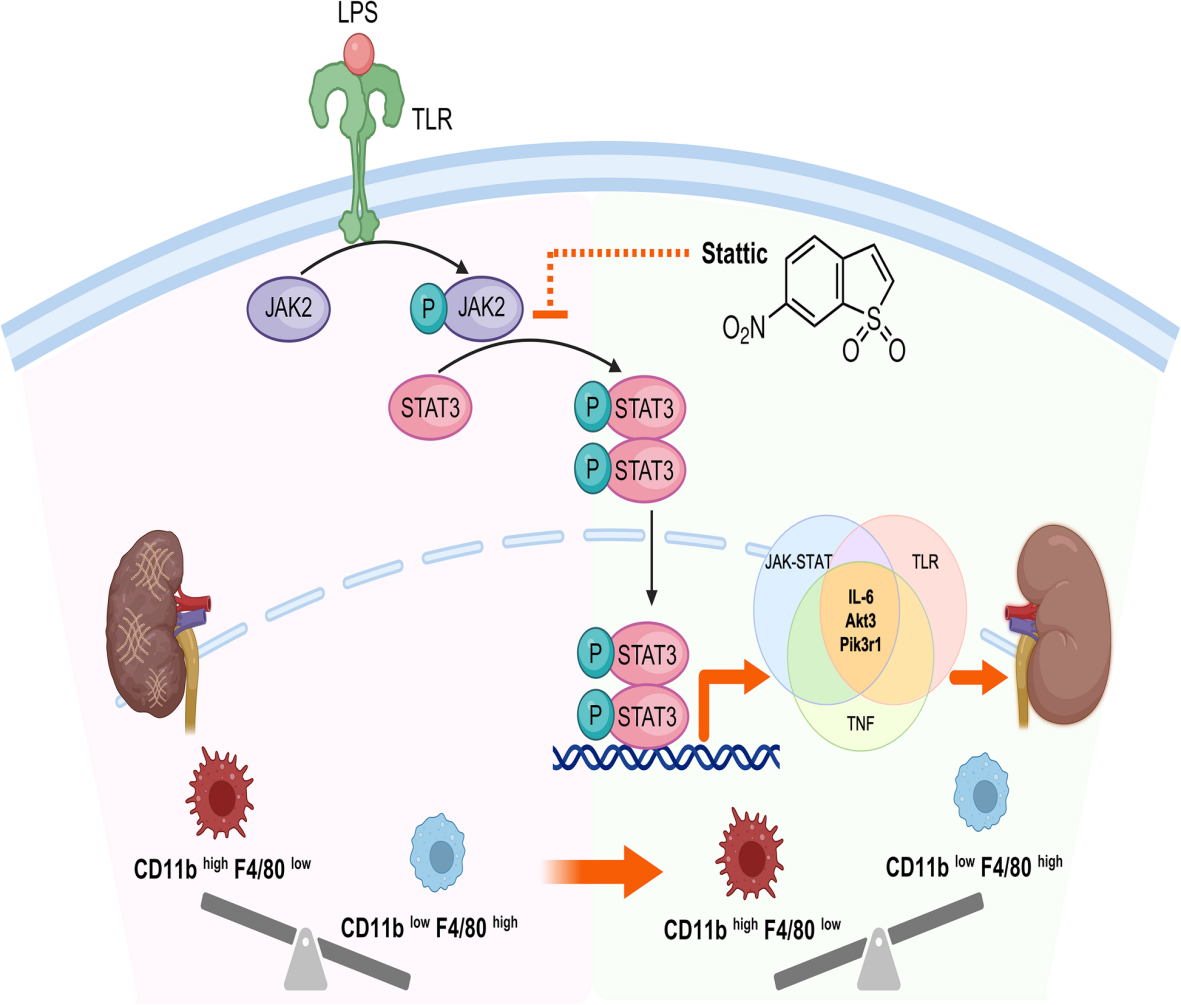
A schematic illustration demonstrating the therapeutic effect of Stattic on LPS-induced kidney injury. Stattic mitigates LPS-induced kidney injury by altering the macrophage subpopulation and downregulating the expression of *IL-6*, *Akt3*, and *Pik3r1*

In this study, we investigated the pathophysiology of L-AKI and explored the therapeutic potential of Stattic, a STAT3 inhibitor, in an L-AKI mouse model. First, our findings highlight the progression of kidney damage and the transition between CD11b^high^F4/80^low^ and CD11b^low^F4/80^high^ macrophage populations following the time-dependent administration of LPS. The most notable changes were observed 6 h after LPS injection, with CD11b^high^F4/80^low^ macrophages showing greater activation of STAT3 than their CD11b^low^F4/80^high^ counterparts. Second, through Stattic treatment, we confirmed the reversal of kidney inflammation and the regulation of macrophage movement, contributing to the suppression of L-AKI. Third, through transcriptomic analysis, we elucidated the mechanism underlying the therapeutic effects of STAT3 inhibitor and identified essential regulatory genes, such as IL-6, Akt3, and Pik3r1, with alternations observed in pro-inflammatory and kidney injury gene profiles. Fourth, Stattic shifted the molecular signatures of macrophages, and the co-culture experiment indicated that LPS-stimulated macrophages are the primary target for STAT3 blockade. Lastly, Stattic demonstrated an anti-fibrotic role following the prolonged LPS exposure in vivo (Fig. 8). For the first time, we identified the pathway modulated by STAT3 in L-AKI mice. Collectively, blockade of the STAT3 signaling pathway decreased in the mRNA expression of genes linked to the PI3K-AKT pathway, as well as IL-6.

Notably, we identified specific macrophage subpopulations characterized by CD11b^high^F4/80^low^ and CD11b^low^F4/80^high^ expression as key players in the exacerbation of L-AKI. In contrast to previous L-AKI model studies that confirmed an increase in F4/80^+^ macrophage levels in the kidney using IHC analysis [27], we used FACS to specifically analyze two macrophage subpopulations: CD11b^high^F4/80^low^ and CD11b^low^F4/80^high^. Our study is distinct from conventional approaches in that it provides a detailed analysis of immune cell phenotypes in the L-AKI model.

Stattic treatment not only restored the balance of macrophage subpopulations but also reduced the expression of inflammatory markers, such as ICAM-1 and pSTAT3. Histological analysis validated these findings, indicating a notable reduction in ICAM-1, NGAL, and pSTAT3 expression after Stattic treatment. This aligns with previous reports suggesting that the progression of kidney injury is associated with inflammatory signaling, particularly through the STAT3 pathway [37].

To elucidate the signal transduction pathways involved in the pathogenesis of L-AKI, we analyzed the transcriptomic profiles of kidneys from mice treated with LPS + Stattic compared to the LPS group. Toll-like receptors (TLRs), including 13 members in mice (TLRs 1–9 and TLRs 11–13), serve as primary initiation points for LPS recognition, triggering downstream transcription factors that lead to the expression of various proinflammatory cytokines, such as TNF-α and IL-6 [38, 39]. Our analysis revealed, for the first time, interconnections among the JAK-STAT, TLR, and TNF signaling pathways, with notable downregulation of *IL-6*, *Akt3*, and *Pik3r1*, key regulators of the PI3K-AKT pathway [40]. In the JAK-STAT signaling pathways, canonical and noncanonical pathways exhibit distinct responses to external stimuli [41]. In our study, Stattic treatment in L-AKI mouse alters the noncanonical pathways related to cell proliferation and survival gene profiles, including *Raf1* (RAF proto-oncogene serine/threonine-protein kinase; MAPK signaling pathway) and *mTOR* (Mechanistic target of rapamycin kinase; PI3K-AKT signaling pathway) [2, 42]. Notably, blocking the STAT3 also influenced *Myc* (MYC proto-oncogene, bHLH transcription factor) [43], which is critical for STAT3-dependent cell-cycle progression. This finding is particularly significant since previous studies have demonstrated that c-Myc promotes tubular apoptosis in AKI [44] and contributes to renal fibrosis following TGF-β treatment [45]. Consistent with these observations, KEGG pathway analysis of Stattic's pluripotency effects revealed its association with 11 genes common to both the response to LPS and macrophage-related genes, highlighting its role in inhibiting the chemokine signaling pathway. This inhibition disrupts cytokine–cytokine receptor interactions, particularly by inactivating *Ccl2* and *Cx3cl1*, which suggests that Stattic effectively alleviates L-AKI symptoms by suppressing the chemokine-dependent JAK-STAT signaling pathway and modulating the activity of innate immune cells, particularly macrophages. Our results provide important evidence that STAT3 blockers can protect renal cells by increasing their survivability and delaying fibrosis progression, highlighting their potential as therapeutic agents in renal diseases.

In transcriptomic validation, the conflicting expression patterns of M2-related CD206 suggest a complex interplay between different macrophage phenotypes during AKI progression. Moreover, we found that the percentage of CD206-positive cells was elevated in the CD11b^low^F4/80^high^ population following Stattic treatment in the L-AKI model. CD206 is a characteristic surface marker of M2-type macrophages, which form most macrophage subsets during short-term recovery [46]. The increase in the percentage of CD206-positive cells in the CD11b^low^F4/80^high^ population upon Stattic treatment suggests an augmentation of M2-type macrophages within that population. M2 macrophages are involved in promoting tissue repair and removing apoptotic cells and debris [47]. Several studies have reported that inhibition of STAT3 encourages polarization toward M2-type macrophages [48, 49]. Taken together, we speculate that the enhanced portion of CD206-positive macrophages observed after Stattic treatment in the CD11b^low^F4/80^high^ population contributes to tissue repair in response to LPS-induced kidney injury. Further detailed studies on the effect of Stattic treatment on macrophage polarization in a L-AKI model are warranted to better understand the immune cell phenotype. On the other hand, excessive activation of STAT3 in the malignant tumor microenvironment has been suggested to induce immunosuppression [50, 51]. This function contrasts with our findings, in which LPS-activated STAT3 rapidly triggered inflammation, leading to kidney injury, and its inhibition alleviated this effect. STAT3 is a critical factor that promotes both proinflammatory and anti-inflammatory responses. This contradictory function is attributed to the acute injury induced by our L-AKI model, which is distinct from the chronically evolving tumor microenvironment [52].

We identified genes responsive to Stattic, such as *IL-6*, *Akt3*, and *Pik3r1*, which play roles in exacerbating L-AKI. However, we did not perform a detailed mechanistic analysis of these genes. Recognizing the limitations of our current research, several key areas for future studies emerge. First, due to STAT3’s widespread expression in renal and immune cells, and our reliance on total RNA expression analysis, it is imperative to further investigate STAT3’s cell-specific roles in the L-AKI model to fully understand its diverse functions. This effort should include an exploration of the dynamics of intracellular communication and its impact on L-AKI progression. Employing single-cell RNA-seq is important for achieving cell type-specific annotation of STAT3 expression levels, which will further expand our understanding. Second, the therapeutic efficacy of specific PI3K inhibitors remains to be evaluated, especially since our study indicated that 3 core genes within the PI3K-Akt-mTOR pathway were downregulated in the LPS + Stattic-treated murine model. Given that a PI3K delta-specific inhibitor has already been approved by the United States Food and Drug Administration for use in anticancer therapy [53], investigating the inhibition of the PI3K signaling pathway in the L-AKI model will provide valuable insights and elucidate the roles of these genes in the transition from AKI to fibrosis, facilitating the identification of novel therapeutic targets and elucidating mechanisms underlying kidney disease.

We measured the complex environment within the kidneys of L-AKI mice. To further investigate macrophage-specific changes, we analyzed whether inflammation induced by LPS in RAW264.7 cells could be inhibited by STAT3 inhibition. It already was reported that LPS treatment activates the inflammation of RAW264.7 cells, accompanied with enhanced iNOS expression [54, 55]. Through FACS analysis, we confirmed that LPS stimulation increased iNOS-positive cells, and these cells were reduced by Stattic treatment. Simultaneously, the pSTAT3^+^iNOS^+^ population was markedly suppressed by Stattic treatment. Based on these results, we inferred that the inflammation in STAT3-activated macrophages is suppressed by STAT3 inhibitor in L-AKI mice. Aligned with this finding, in a co-culture system of macrophages and tubular epithelial cells, we observed that LPS-stimulated RAW264.7 cells caused tubular epithelial cell injury, even without direct LPS exposure to OK cells. STAT3 blockade attenuated this response, supporting the potential of STAT3 inhibition to reduce macrophage-specific inflammatory responses.

Additionally, we investigated the fibrotic changes in the kidney induced by LPS. In Our L-CKD experiment, the expression of fibrosis markers such as fibronectin and COLA1 was increased in the kidney, along with enhanced collagen accumulation, which was also confirmed by tissue staining. This fibrosis was ameliorated by Stattic treatment. Interestingly, we observed opposing patterns in the transcriptomic analysis of the M2 macrophage marker CD206 between L-AKI and L-CKD mice. This contrast is likely due to the considerably different internal environments between acute and chronic kidney injury. While M2 macrophages play a role in reprogramming and protecting kidney tissue during acute injury, they promote fibrosis by secreting TGF-β in chronic kidney disease [8]. Our study found that STAT3 inhibition exhibited anti-fibrotic effects even when kidney fibrosis had progressed. We confirmed that this result is consistent with our previous findings, which reported that Stattic treatment alleviated unilateral IRI-induced chronic kidney disease, though not LPS-induced [22]. Furthermore, unlike in L-AKI, there were no changes in the mRNA expression of *Akt3* and *Pik3r1* in L-CKD, and we plan to conduct further pathway mechanism studies, such as RNA sequencing, in the future study.

## Conclusions

In summary, this study provides comprehensive insights into the progression of L-AKI and highlights the therapeutic potential of Stattic. Transcriptome analysis of the L-AKI model revealed a reduction in the mRNA expression levels of *IL-6*, *Akt3*, and *Pik3r1* induced by LPS stimulation following Stattic treatment. Additionally, dynamic transitions in macrophage phenotypes were observed in the L-AKI model, and the increase in CD206-positive cells within the CD11b^low^F4/80^high^ population upon Stattic treatment was particularly intriguing. This study elucidated the pathophysiologic alterations associated with L-AKI and the protective effects of STAT3 inhibition. We also confirmed the anti-fibrotic effects of Stattic treatment in L-CKD. Overall, STAT3 plays a major role in macrophage plasticity and contributes to L-AKI and L-CKD development. Taken together, STAT3 blockade serves as an important therapeutic intervention agent by exhibiting anti-inflammatory and anti-fibrotic effects, thereby alleviating LPS-induced kidney disease.

## Supplementary Information

Supplementary Material 1: Figure S1. Representative IHC images of pSTAT3 in kidney tubules and transcriptomic analysis of time-dependent L-AKI mice. (A) pSTAT3 staining of the L-AKI model following a time-dependent manner (0, 6, 12, and 24 h) is shown. (B) The expression of pSTAT3 decreased in the LPS + Stattic group compared to that in the LPS group. Scale bar, 75 μm (200X). (C) The mRNA expression of kidney injury marker, pro-inflammatory, STAT3-associated genes in time-dependent L-AKI mice. Figure S2. Top 10 KEGG pathways and GO enrichment analyses for mice treated with LPS and Stattic. (A, B) Top 10 global KEGG pathways and GO enrichment analyses were conducted for both downregulated (A) and upregulated (B) genes. The results are visualized in dot plots, where the dot size corresponds to the gene count, and the color gradient represents the *p*-value . Figure S3. Common genes derived from JAK-STAT-dependent inflammatory response pathways. (A) Common genes across three distinct inflammatory response pathways were identified using KEGG pathway analysis. (B) A heatmap was constructed to visualize macrophage-related genes, specifically highlighting differences in macrophage cytokine production, activation, and migration. Figure S4. KEGG pathway analysis on the three distinct inflammatory response pathways. KEGG pathway analysis was performed on three distinct inflammatory response pathways: JAK-STAT, TLR, and TNF, represented in blue, red, and green, respectively. Additionally, 3 genes common to all pathways were further analyzed and visualized using the KEGG color map. Figure S5. KEGG pathway analysis of the response to LPS and macrophage-related genes. (A) Color maps depicting the 11 intersecting genes identified between the response to LPS and macrophage-related gene pathways, focusing on the cytokine–cytokine receptor interaction and chemokine signaling pathways. (B) Detailed KEGG pathway analysis of thermogenesis highlighting upregulated gene interactions. Figure S6. JAK-STAT signaling pathway-related upregulated genes and KEGG pathway analysis. (A) A heatmap represented JAK-STAT signaling pathway-related upregulated genes (*n*  = 4). (B) A dot plot for KEGG analysis of upregulated and downregulated genes was shown. (C) A table lists IL-6-mediated STAT3 signaling pathway-related genes identified from the KEGG pathway analysis. (D) A diagram from the KEGG mapper indicated both canonical (gray line) and noncanonical (red line) STAT signaling pathways, along with upregulated (red) and downregulated (green) genes. (E) A network analysis of the IL-6-mediated STAT3 signaling pathway was derived from (C). Figure S7. Network analysis of GO terms related to LPS and macrophage genes. A comprehensive network analysis of LPS and macrophage-related genes was constructed using Cytoscape software. Figure S8. Systemic changes in the spleens of L-AKI mice using real-time qPCR. The expression levels of (A) proinflammatory genes and (B) macrophage-related and STAT3-associated genes were analyzed in the spleens of L-AKI mice (*n*  = 8 in each group). The results are presented as mean ± SEM, and statistical analysis was conducted using an unpaired two-tailed Student’s t-test. * *P*  < 0.05, ** *P*  < 0.01, *** *P*  < 0.001. Figure S9. pSTAT3-positive cells of LPS-stimulated RAW264.7 cells. Histogram and quantification graph of pSTAT3-positive cells using FACS analysis after (A) time-dependent, (B) dose-dependent LPS treatment, and (C) Stattic treatment with LPS stimulation in RAW264.7 cells.

## Data Availability

All data and materials are available from the corresponding authors upon a reasonable request.

## Abbreviations

STAT3: Signal transducer and activator of transcription 3
AKI: Acute kidney injury
LPS: Lipopolysaccharide
L-AKI: Lipopolysaccharide-induced acute kidney injury
TLR: Toll-like receptor
iNOS: Inducible nitric oxide synthase
BUN: Blood urea nitrogen
IHC: Immunohistochemistry
PAS: Periodic Acid-Schiff
DEGs: Differentially expressed genes
KEGG: Kyoto Encyclopedia of Genes and Genomes
GO: Gene Ontology
L-CKD: Lipopolysaccharide-induced chronic kidney disease
SEM: Standard error of the mean

## Data availability

No datasets were generated or analysed during the current study.
