# Supplementary material for: STAT3 blockade ameliorates LPS-induced kidney injury through macrophage-driven inflammation
Source: Cell Commun Signal. 2024 Oct 4;22:476. doi: 10.1186/s12964-024-01841-1 (PMC11453053; doi:10.1186/s12964-024-01841-1)
Supplement: Supplementary file 1 — Supplementary Material 1: Figure S1. Representative IHC images of pSTAT3 in kidney tubules and transcriptomic analysis of time-dependent L-AKI mice. (A) pSTAT3 staining of the L-AKI model following a time-dependent manner (0, 6, 12, and 24 h) is shown. (B) The expression of pSTAT3 decreased in the LPS + Stattic group compared to that in the LPS group. Scale bar, 75 μm (200X). (C) The mRNA expression of kidney injury marker, pro-inflammatory, STAT3-associated genes in time-dependent L-AKI mice. Figure S2. Top 10 KEGG pathways and GO enrichment analyses for mice treated with LPS and Stattic. (A, B) Top 10 global KEGG pathways and GO enrichment analyses were conducted for both downregulated (A) and upregulated (B) genes. The results are visualized in dot plots, where the dot size corresponds to the gene count, and the color gradient represents the p-value . Figure S3. Common genes derived from JAK-STAT-dependent inflammatory response pathways. (A) Common genes across three distinct inflammatory response pathways were identified using KEGG pathway analysis. (B) A heatmap was constructed to visualize macrophage-related genes, specifically highlighting differences in macrophage cytokine production, activation, and migration. Figure S4. KEGG pathway analysis on the three distinct inflammatory response pathways. KEGG pathway analysis was performed on three distinct inflammatory response pathways: JAK-STAT, TLR, and TNF, represented in blue, red, and green, respectively. Additionally, 3 genes common to all pathways were further analyzed and visualized using the KEGG color map. Figure S5. KEGG pathway analysis of the response to LPS and macrophage-related genes. (A) Color maps depicting the 11 intersecting genes identified between the response to LPS and macrophage-related gene pathways, focusing on the cytokine–cytokine receptor interaction and chemokine signaling pathways. (B) Detailed KEGG pathway analysis of thermogenesis highlighting upregulated gene interactions. Figure [file 12964_2024_1841_MOESM1_ESM.pdf]

Supplementary materials

Figure S1. Representative IHC images of pSTAT3 in kidney tubules and transcriptomic analysis of time-dependent L-AKI mice

Figure S2. Top 10 KEGG pathways and GO enrichment analyses for mice treated with LPS and Stattic

Figure S3. Common genes derived from JAK-STAT-dependent inflammatory response pathways

Figure S4. KEGG pathway analysis on the three distinct inflammatory response pathways

Figure S5. KEGG pathway analysis of response to LPS and macrophage-related genes

Figure S6. JAK-STAT signaling pathway-related upregulated genes and KEGG pathway analysis

Figure S7. Network analysis of GO terms related to LPS and macrophage-related genes

Figure S8. Systemic changes in the spleens of L-AKI mice using real-time qPCR

Figure S9. pSTAT3-positive cells of LPS-stimulated RAW264.7 cells

Figure S1.

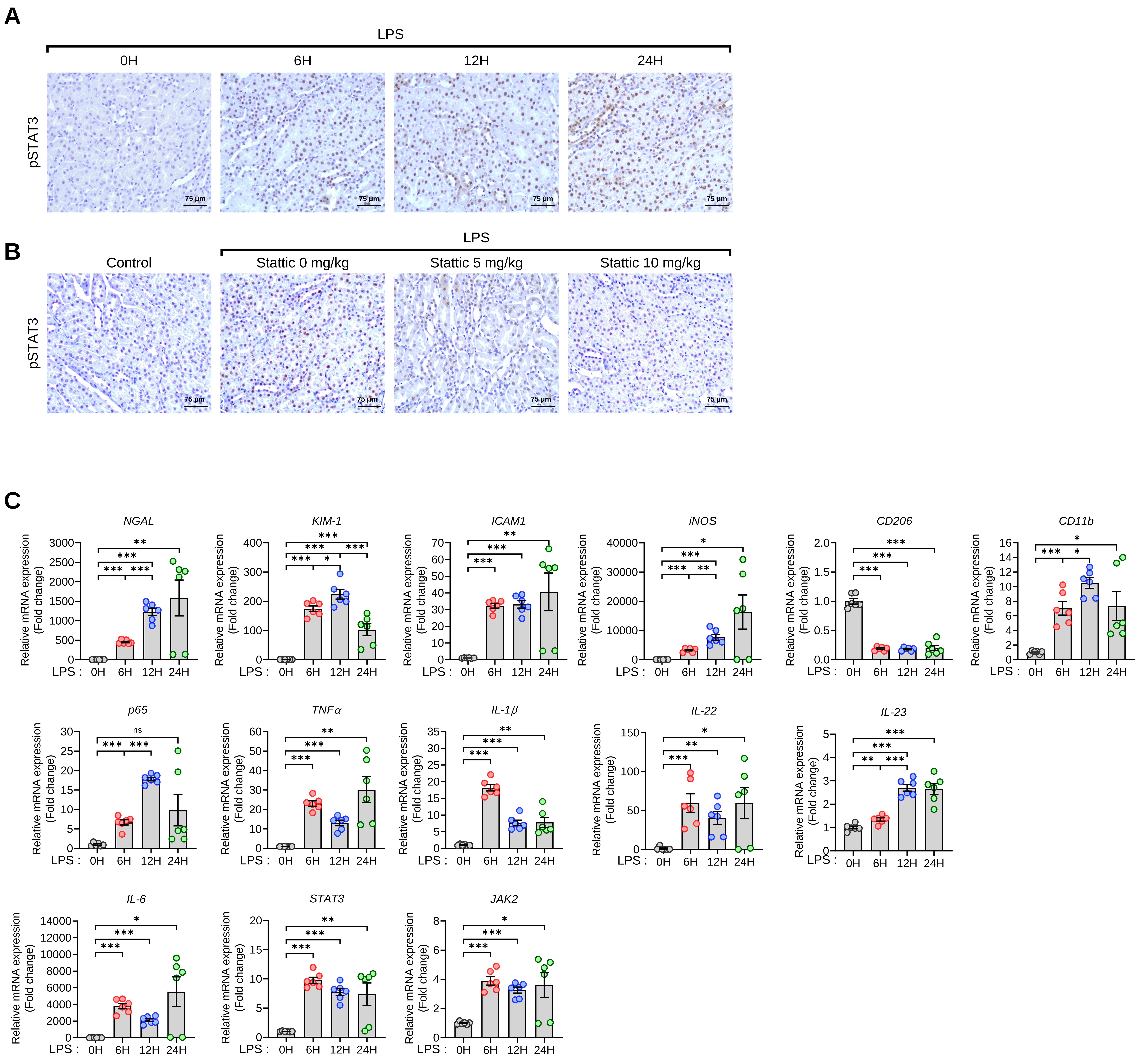

Figure S1. Representative IHC images of pSTAT3 in kidney tubules and transcriptomic analysis of time-dependent L-AKI mice

(A) pSTAT3 staining of the L-AKI model following a time-dependent manner (0, 6, 12, and 24 h) is shown. (B) The expression of pSTAT3 decreased in the LPS + Stattic group compared to that in the LPS group. Scale bar, 75 μm (200X). (C) The mRNA expression of kidney injury marker, pro-inflammatory, STAT3-associated genes in time-dependent L-AKI mice.

Figure S2.

A

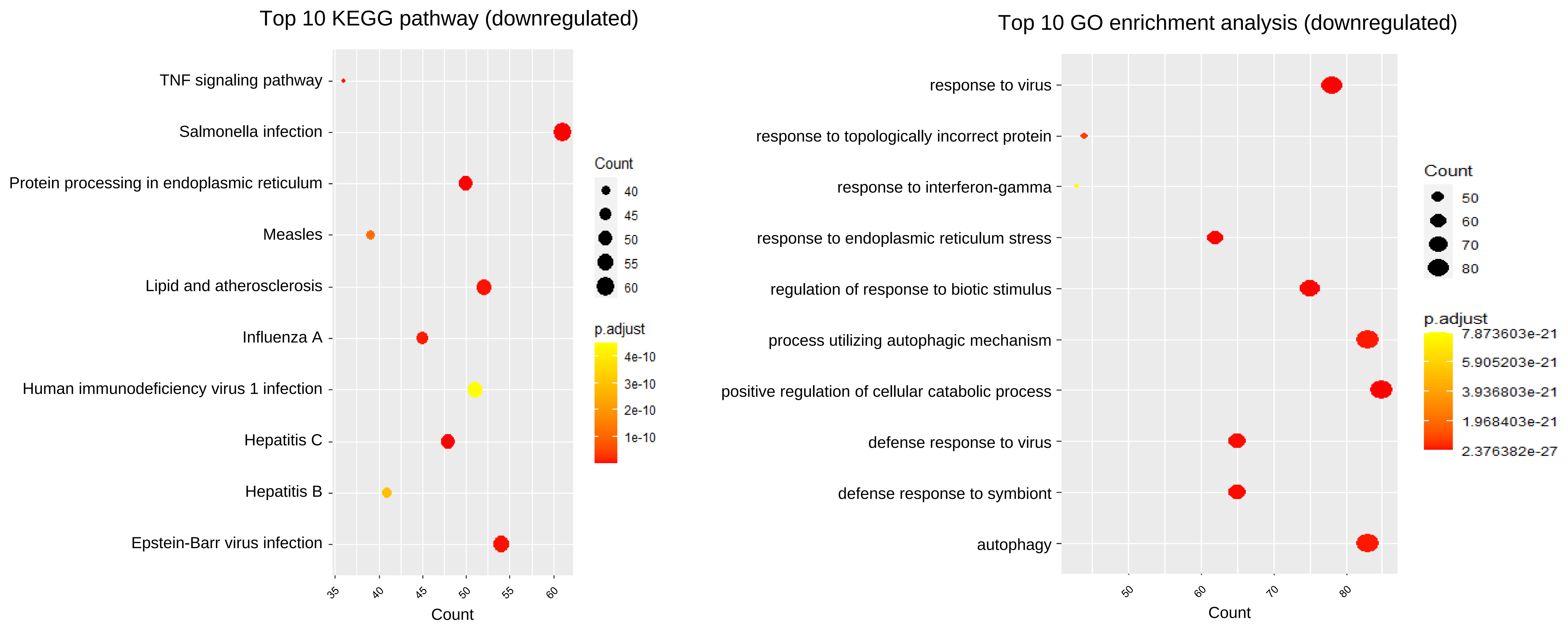

B

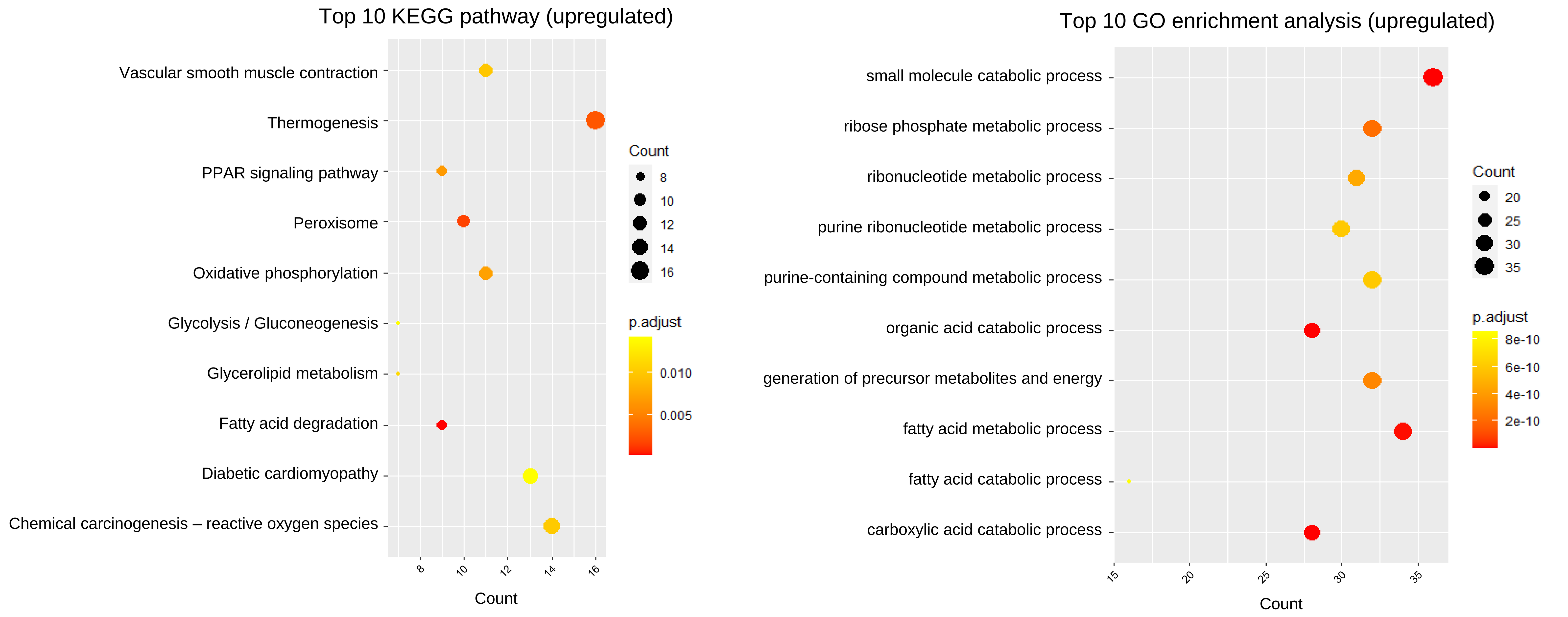

Figure S2. Top 10 KEGG pathways and GO enrichment analyses for mice treated with LPS and Stattic
(A, B) Top 10 global KEGG pathways and GO enrichment analyses were conducted for both downregulated (A) and upregulated (B) genes. The results are visualized in dot plots, where the dot size corresponds to the gene count, and the color gradient represents the *p-value*.

Figure S3.

A

Name	Log$_2$FC	*p*-value
Stat1	-0.817	0.012
Ifnar2	-0.551	0.032
Ifnar1	-0.532	0.044
Jak1	-0.417	0.013

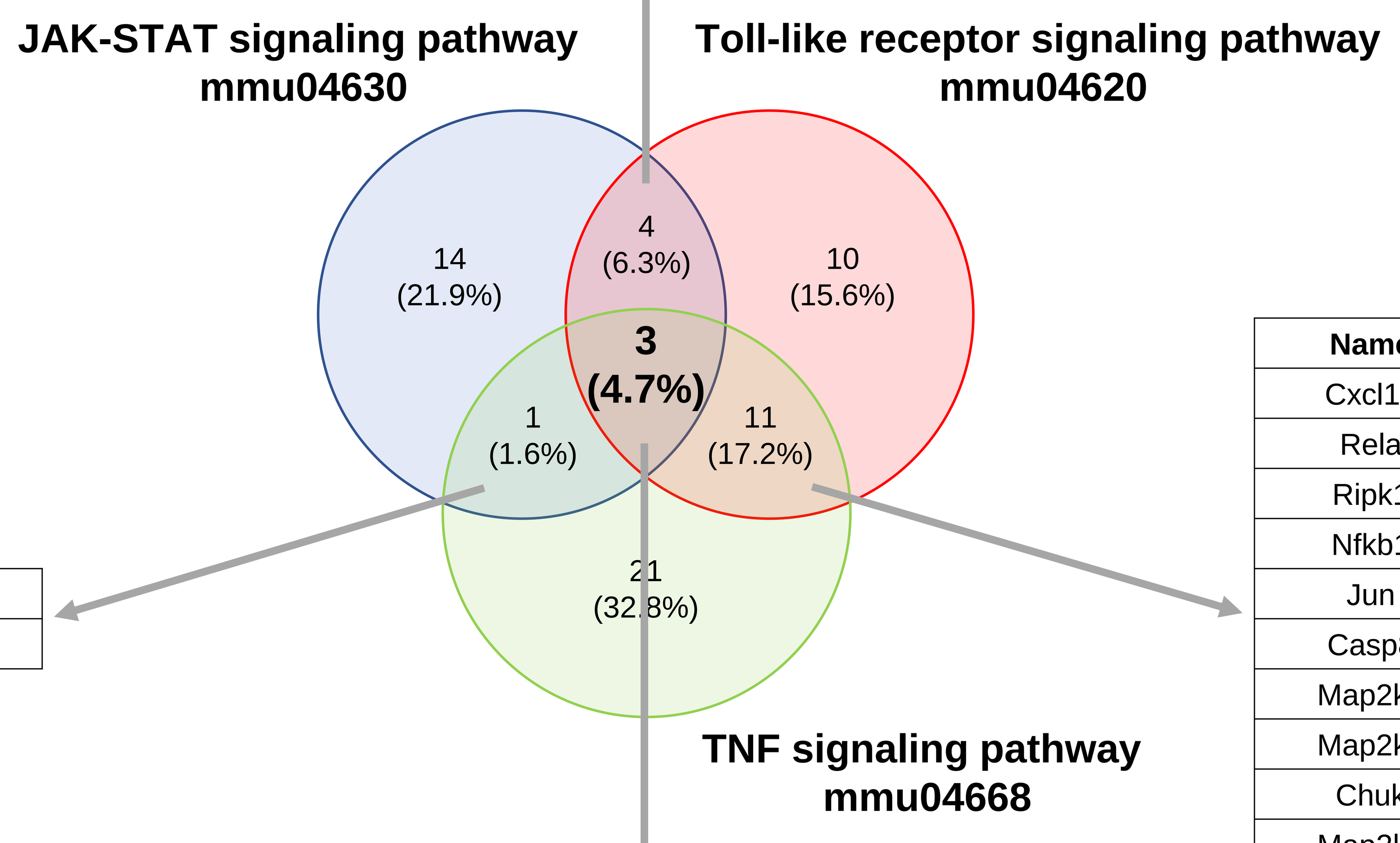

Name	Log$_2$FC	*p*-value
Socs3	-1.605	0.023

Name	Log$_2$FC	*p*-value
Cxcl10	-2.507	0.004
Rela	-1.299	0.004
Ripk1	-0.939	0.012
Nfkb1	-0.872	0.005
Jun	-0.868	0.037
Casp8	-0.838	0.020
Map2k1	-0.404	0.039
Map2k4	-0.300	0.049
Chuk	-0.299	0.036
Map2k3	-0.297	0.025
Ikbkg	-0.230	0.011

Name	Log$_2$FC	*p*-value
IL-6	-1.54277	0.018
Akt3	-0.89794	0.035
Pik3r1	-0.83743	0.009

B

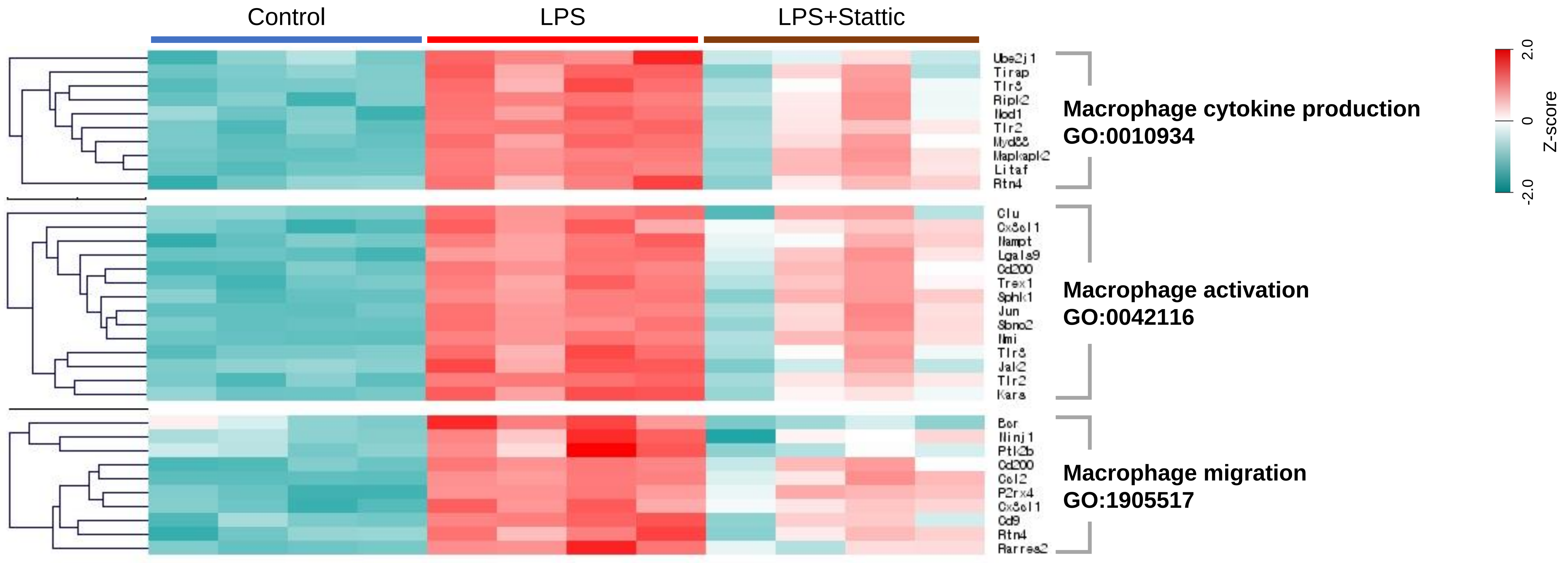

Figure S3. Common genes derived from JAK-STAT-dependent inflammatory response pathways
(A) Common genes across three distinct inflammatory response pathways were identified using KEGG pathway analysis. (B) A heatmap was constructed to visualize macrophage-related genes, specifically highlighting differences in macrophage cytokine production, activation, and migration.

Figure S4.

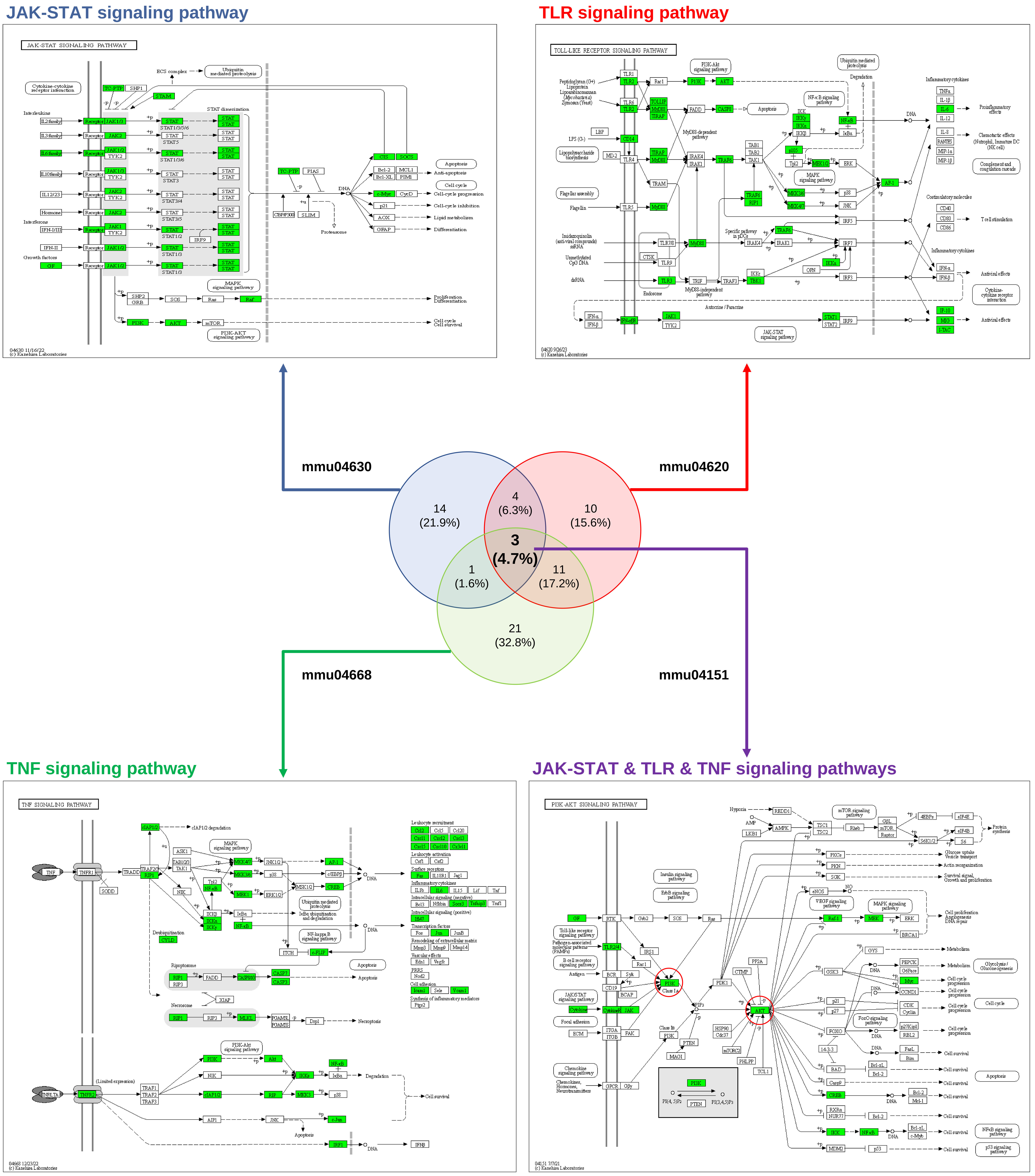

Figure S4. KEGG pathway analysis on the three distinct inflammatory response pathways

KEGG pathway analysis was performed on three distinct inflammatory response pathways: JAK-STAT, TLR, and TNF, represented in blue, red, and green, respectively. Additionally, 3 genes common to all pathways were further analyzed and visualized using the KEGG color map.

Figure S5.

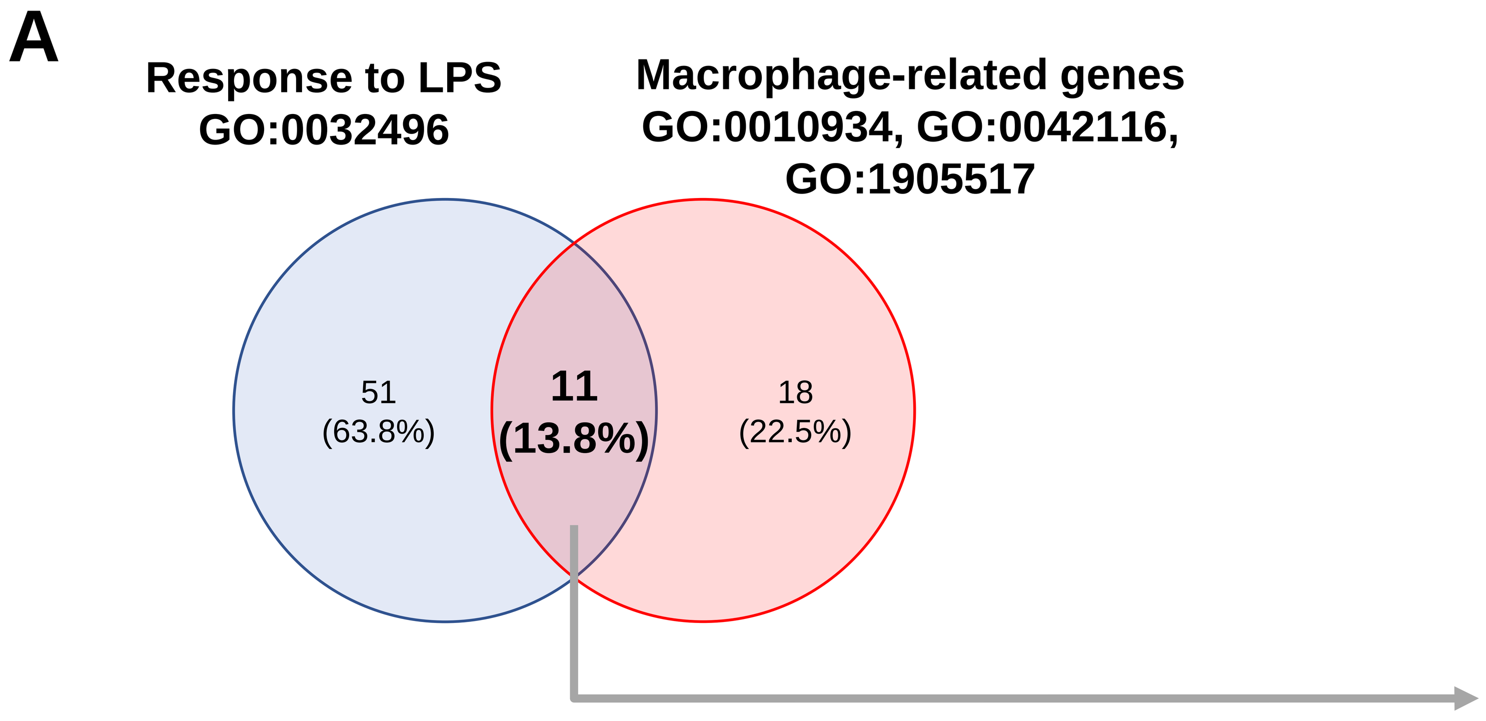

Name	Log_2FC	*p*-value
Ccl2	-1.243	0.034
Jak2	-1.164	0.007
Litaf	-1.147	0.010
Myd88	-1.100	0.012
Sbno2	-0.895	0.031
Tirap	-0.869	0.017
Ripk2	-0.835	0.011
Lgals9	-0.750	0.044
Mapkapk2	-0.722	0.033
Bcr	-0.647	0
Cx3cl1	-0.494	0.008

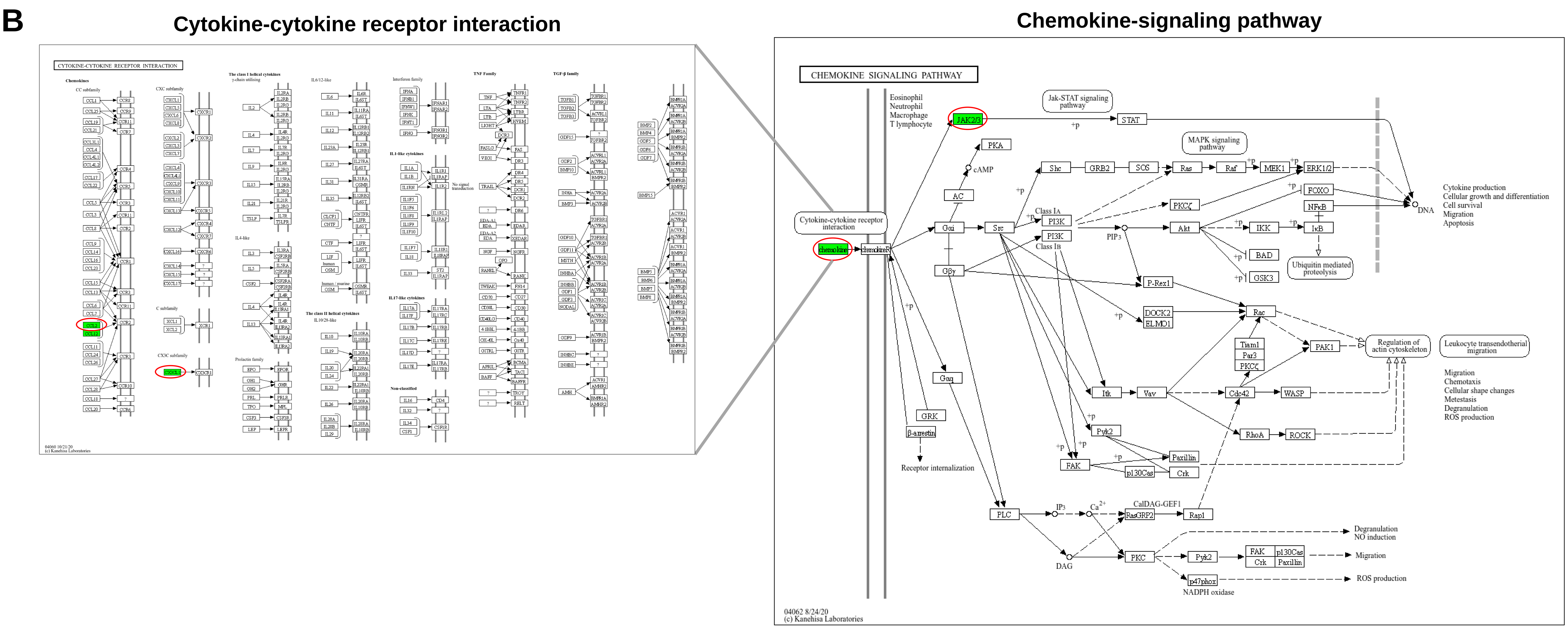

Figure S5. KEGG pathway analysis of the response to LPS and macrophage-related genes

(A) Color maps depicting the 11 intersecting genes identified between the response to LPS and macrophage-related genes pathways, focusing on the cytokine-cytokine receptor interaction and chemokine signaling pathways. (B) Detailed KEGG pathway analysis of thermogenesis highlighting upregulated gene interactions.

Figure S6.

A

JAK-STAT signaling pathway (mmu04630): upregulated genes

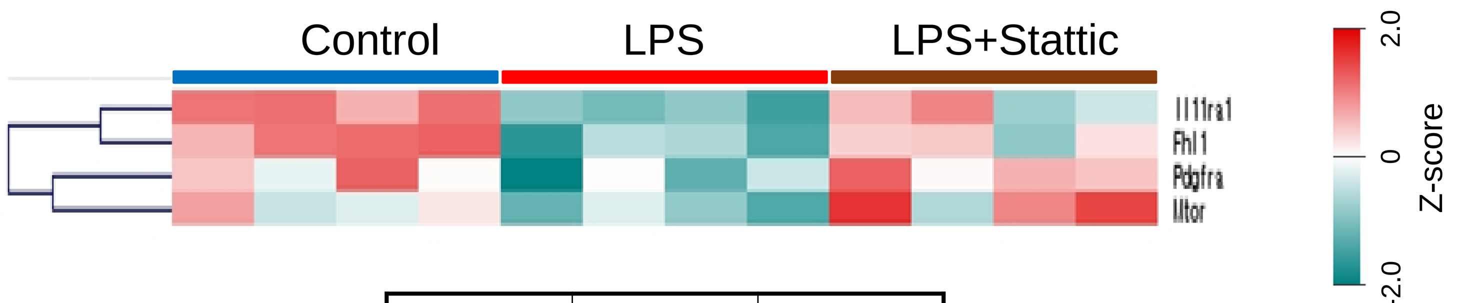

Name	Log_2FC	*p*-value
Pdgfra	0.736	0.022
Il11ra1	0.533	0.046
Fhl1	0.452	0.033
Mtor	0.494	0.022

B

KEGG enrichment analysis (down & upregulation)

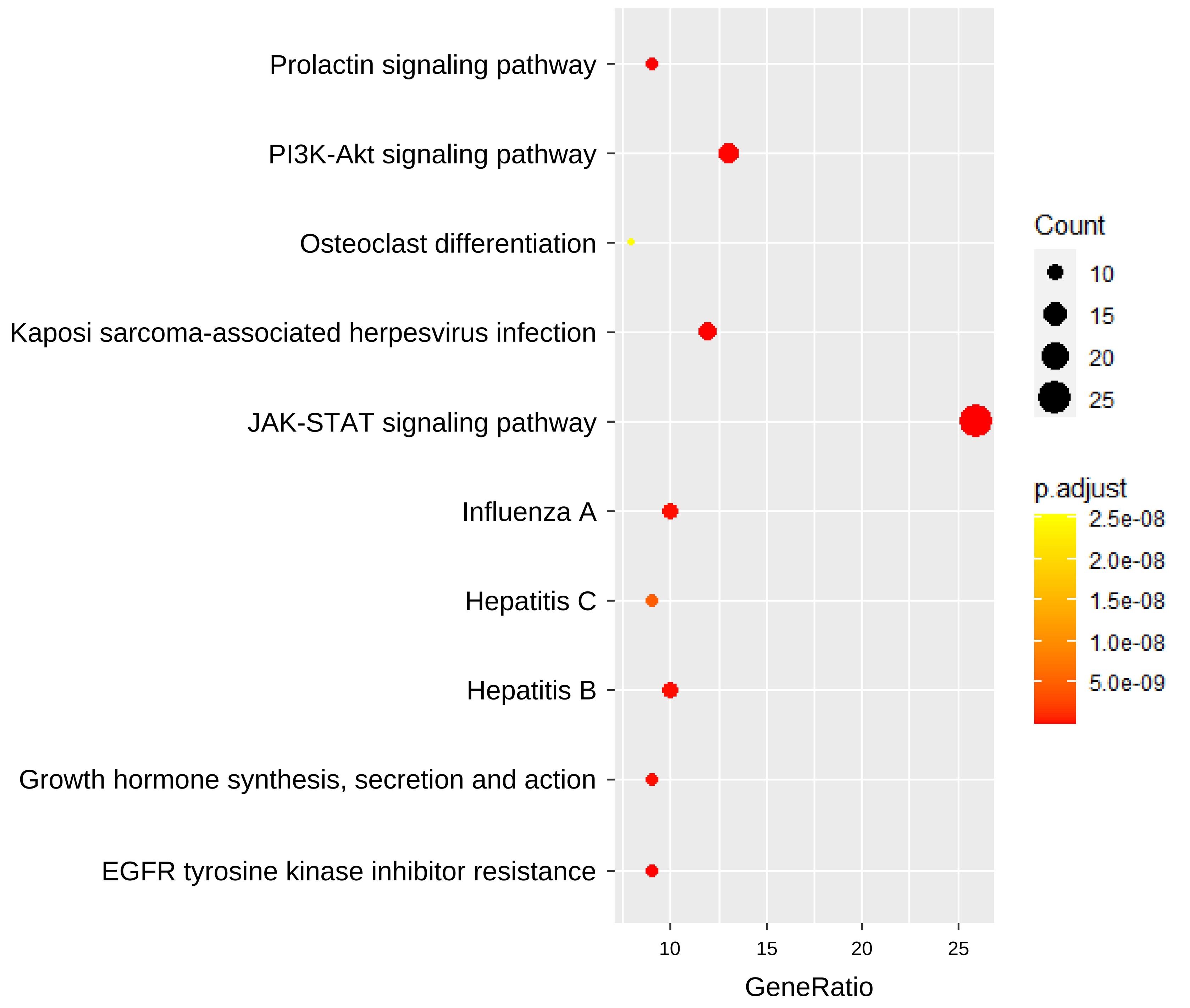

C

IL-6 mediated STAT3 signaling pathway related genes

Name	KEGG ID	Log_2FC	*p*-value
Mtor	mmu:56717	0.495	0.022
Fhl1	mmu:14199	0.452	0.033
Stat6	mmu:20852	-0.307	0.019
Raf1	mmu:110157	-0.316	0.043
Jak1	mmu:16451	-0.417	0.013
Stat3	mmu:20848	-0.713	0.051
Stat1	mmu:20846	-0.817	0.012
Ptpn2	mmu:19255	-0.935	0.013
Myc	mmu:17869	-1.13	0.014
Jak2	mmu:16452	-1.17	0.007
IL-6	mmu:16193	-1.54	0.018

D

JAK-STAT signaling pathway: canonical (gray) vs. noncanonical (red)

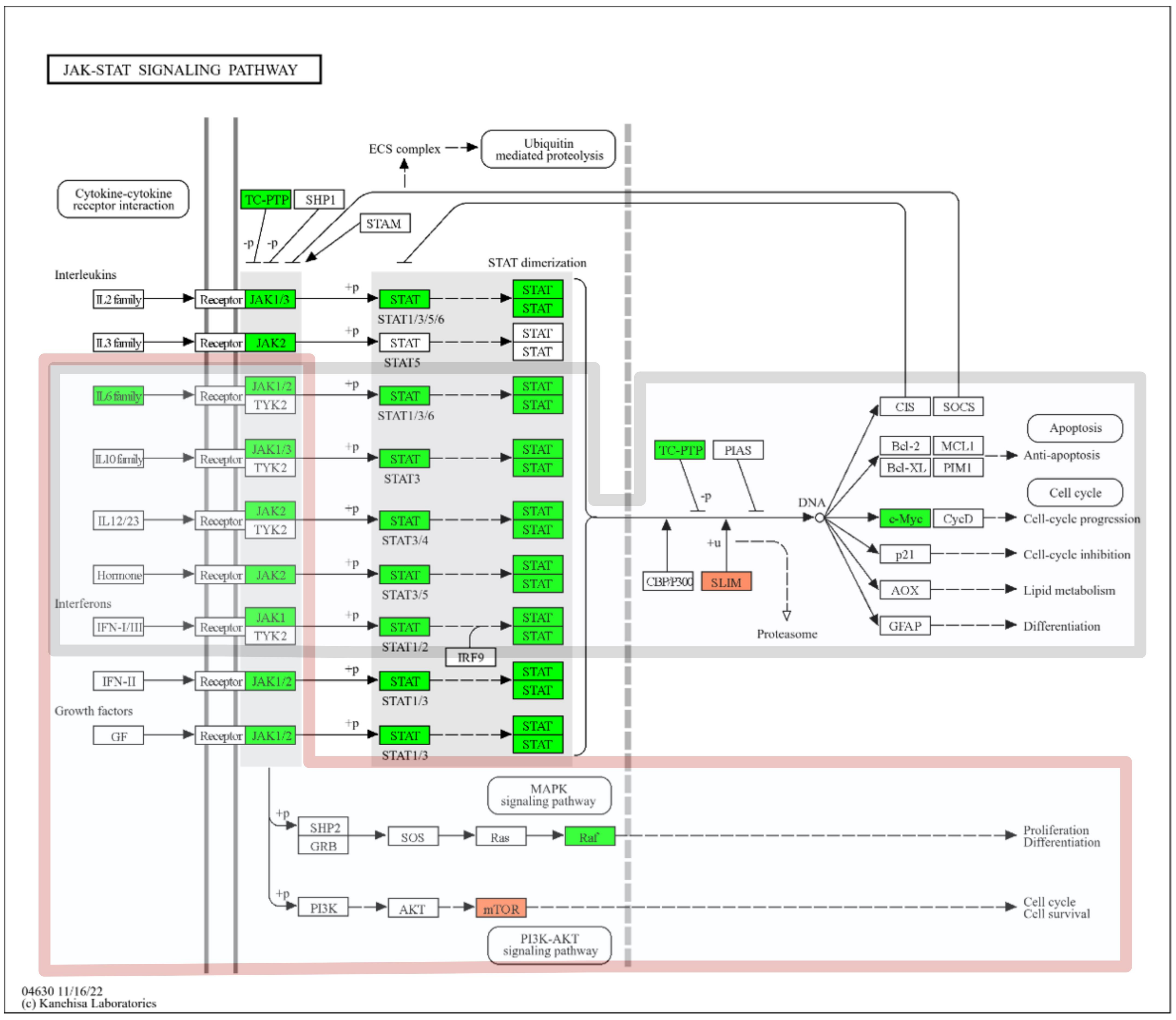

E

IL-6-mediated STAT3 signaling pathway

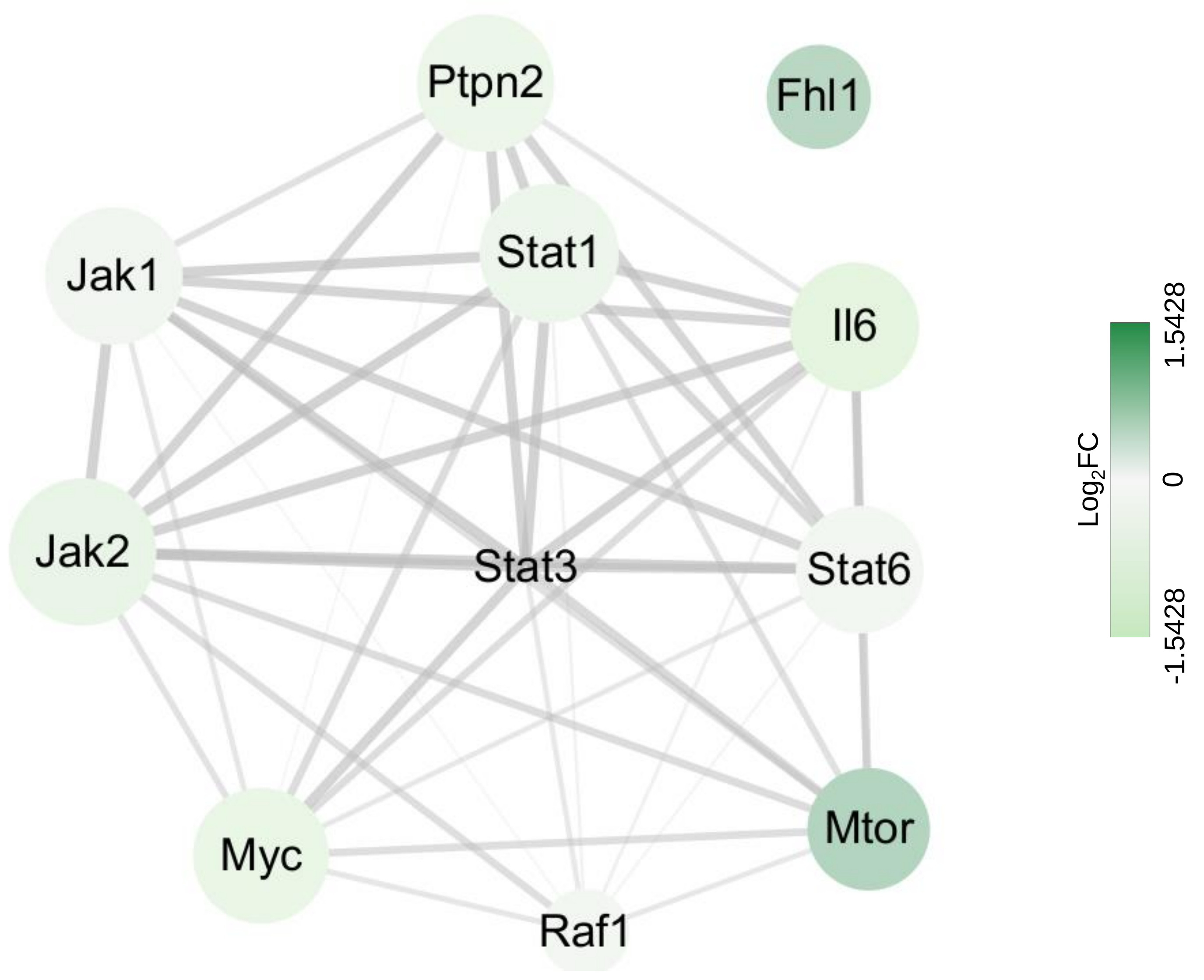

Figure S6. JAK-STAT signaling pathway-related upregulated genes and KEGG pathway analysis (A) A heatmap represented JAK-STAT signaling pathway-related upregulated genes (*n* = 4). (B) A dot plot for KEGG analysis of upregulated and downregulated genes was shown. (C) A table lists IL-6-mediated STAT3 signaling pathway-related genes identified from the KEGG pathway analysis. (D) A diagram from the KEGG mapper indicated both canonical (gray line) and noncanonical (red line) STAT signaling pathways, along with upregulated (red) and downregulated (green) genes. (E) A network analysis of the IL-6-mediated STAT3 signaling pathway was derived from (C).

Figure S7.

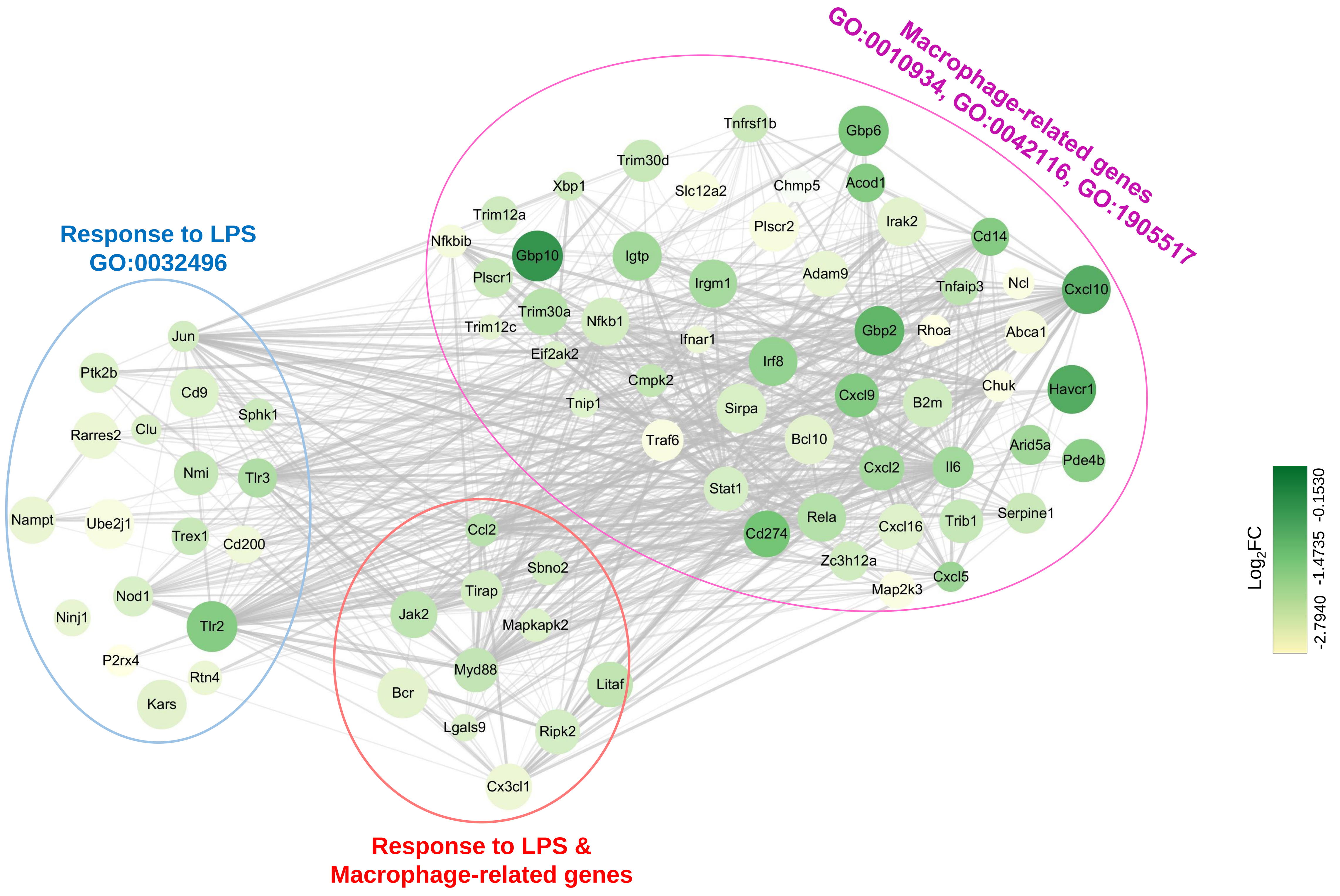

Figure S7. Network analysis of GO terms related to LPS and macrophage genes

A comprehensive network analysis of the response to LPS and macrophage-related genes was constructed using Cytoscape software.

Figure S8.

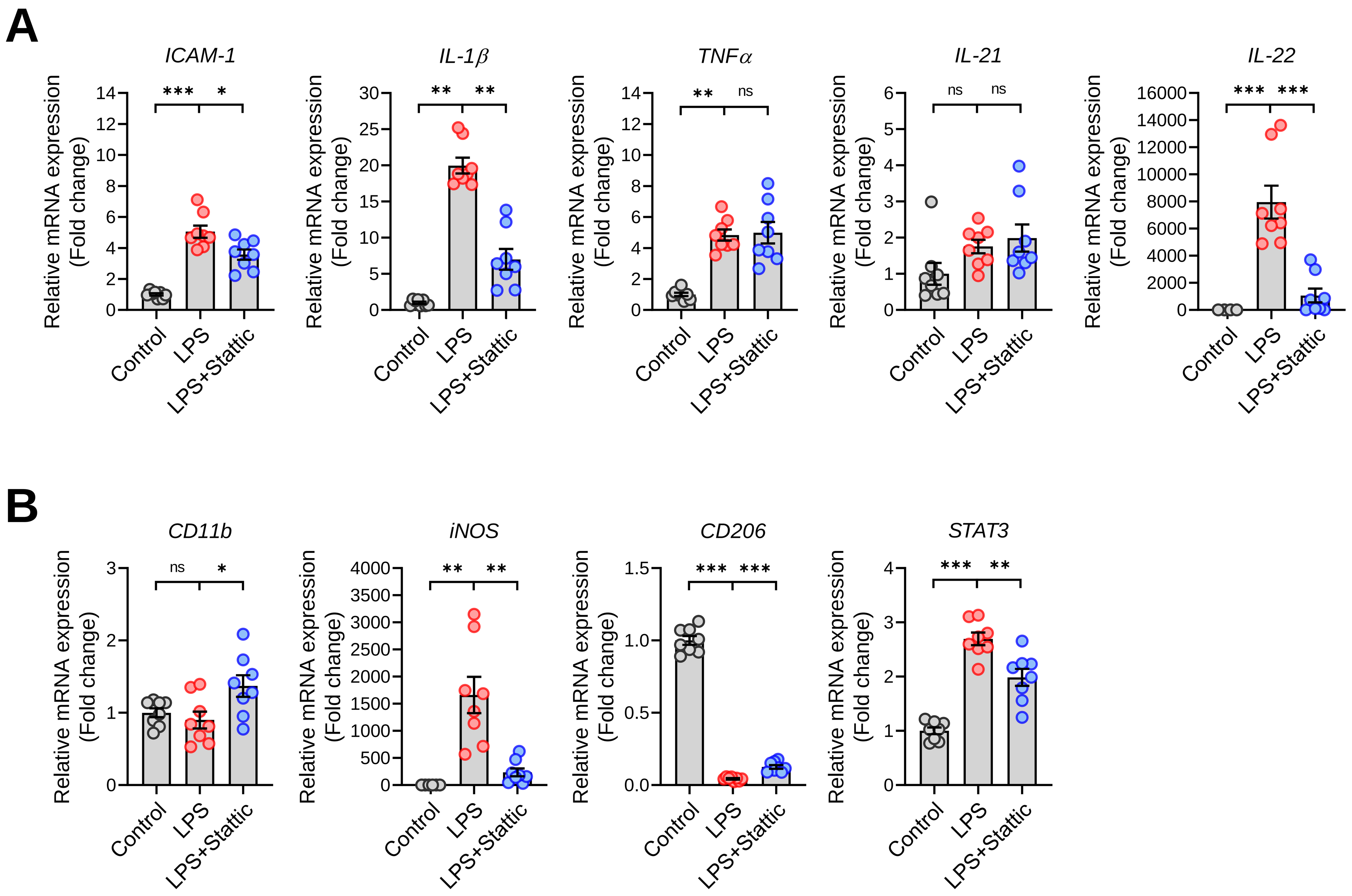

Figure S8. Systemic changes in the spleens of L-AKI mice using real-time qPCR
The expression levels of (A) proinflammatory genes and (B) macrophage-related and STAT3-associated genes were analyzed in the spleens of L-AKI mice (n = 8 in each group). The results are presented as mean ± SEM, and statistical analysis was conducted using an unpaired two-tailed Student's t-test. *P < 0.05, **P < 0.01, ***P < 0.001.

Figure S9.

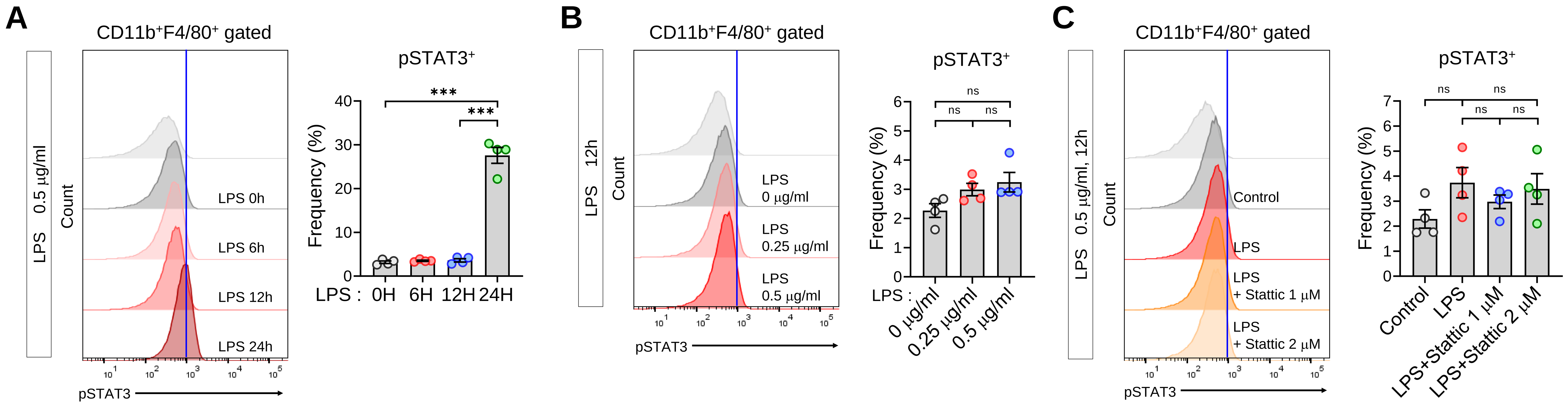

Figure S9. pSTAT3-positive cells of LPS-stimulated RAW264.7 cells

Histogram and quantification graph of pSTAT3-positive cells using FACS analysis after (A) time-dependent, (B) dose-dependent LPS treatment, and (C) Stattic treatment with LPS stimulation in RAW264.7 cells.
